# A randomized trial of mailed outreach with behavioral economic interventions to improve liver cancer surveillance

**DOI:** 10.1097/HC9.0000000000000349

**Published:** 2023-12-15

**Authors:** Shivan J. Mehta, Caitlin McDonald, Catherine Reitz, Shivani Kastuar, Christopher K. Snider, Evelyn Okorie, Kiernan McNelis, Hamzah Shaikh, Tessa S. Cook, David S. Goldberg, Kenneth Rothstein

**Affiliations:** 1 Department of Medicine, Perelman School of Medicine, University of Pennsylvania, Philadelphia, USA; 2 Center for Health Care Innovation, University of Pennsylvania, Philadelphia, USA; 3 Department of Radiology, Perelman School of Medicine, University of Pennsylvania, Philadelphia, USA; 4 Department of Medicine, Miller School of Medicine, University of Miami, Miami, Florida, USA

## Abstract

**Background:**

Surveillance rates for HCC remain limited in patients with cirrhosis. We evaluated whether opt-out mailed outreach increased uptake with or without a $20 unconditional incentive.

**Methods:**

This was a pragmatic randomized controlled trial in an urban academic health system including adult patients with cirrhosis or advanced fibrosis, at least 1 visit to a specialty practice in the past 2 years and no surveillance in the last 7 months. Patients were randomized in a 1:2:2 ratio to (1) usual care, (2) a mailed letter with a signed order for an ultrasound, or (3) a mailed letter with an order and a $20 unconditional incentive. The main outcome was the proportion with completion of ultrasound within 6 months.

**Results:**

Among the 562 patients included, the mean age was 62.1 (SD 11.1); 56.8% were male, 51.1% had Medicare, and 40.6% were Black. At 6 months, 27.6% (95% CI: 19.5–35.7) completed ultrasound in the Usual care arm, 54.5% (95% CI: 47.9–61.0) in the Letter + Order arm, and 54.1% (95% CI: 47.5–60.6) in the Letter + Order + Incentive arm. There was a significant increase in the Letter + Order arm compared to Usual care (absolute difference of 26.9%; 95% CI: 16.5–37.3; *p*<0.001), but no significant increase in the Letter + Order + Incentive arm compared to Letter + Order (absolute difference of −0.4; 95% CI: −9.7 to 8.8; *p*=0.93).

**Conclusions:**

There was an increase in HCC surveillance from mailed outreach with opt-out framing and a signed order slip, but no increase in response to the financial incentive.

## Graphical abstract

**Figure:**
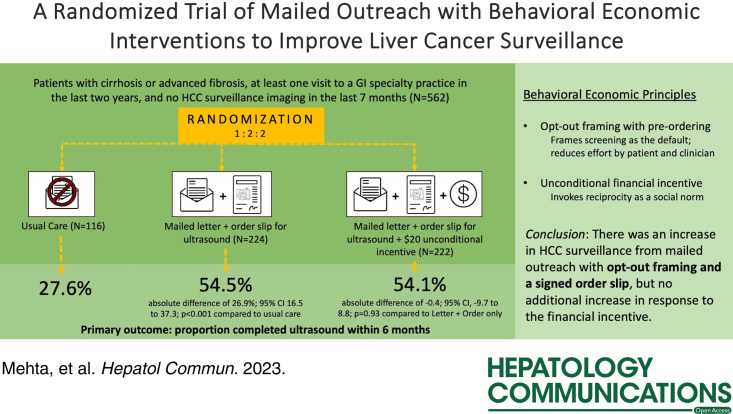

## INTRODUCTION

Hepatocellular carcinoma (HCC) is a leading cause of cancer-related death in the United States, and the vast majority of cases occur in the setting of liver cirrhosis.^1–3^ Outcomes are improved if HCC is treated early, which is why guidelines recommend routine surveillance with abdominal ultrasound every 6 months for patients with cirrhosis.^4–6^ Despite the recognition of this in the guidelines, surveillance rates remain low across different populations.^7–10^ Interventions to increase surveillance rates include patient navigation, mailed reminders, and electronic alerts to clinicians, but they have either have limited effectiveness or have not scaled broadly.^4,11^

Behavioral economics is a relatively new discipline that explores how humans have systematic biases that limit participation in prevention, such as inertia (status-quo bias) or overweighing current costs compared to future benefits (present-time bias).^12–16^ However, the field has shown that these same biases can be harnessed to promote healthy behavior.^17,18^ For example, shifting the framing of participation from opt-in to opt-out has been shown to increase cancer screening and vaccination.^19,20^ Financial incentives have had mixed results when applied to health prevention, but studies have shown that an unconditional incentive can increase participation by invoking reciprocity as a social norm.^21–26^

In this pragmatic trial, we evaluated the effect of opt-out framing with preordering with or without an unconditional $20 incentive on the response to HCC surveillance outreach among patients who are due for screening across a specialty practice.

## METHODS

### Study design

This was a 3-arm randomized controlled trial applying behavioral economic approaches to encourage patients with liver cirrhosis or advanced fibrosis to complete routine surveillance ultrasounds. Eligible patients were randomized in a 1:2:2 ratio into 3 arms: (1) Usual care, (2) Letter + Order, or (3) Letter + Order + Incentive. They were identified in a series of 3 batches each 3 months apart, and randomization was stratified by batch. Patients in the Usual care arm received standard of care and were not mailed any outreach as part of this study. Patients in the Letter + Order arm were sent a letter invoking opt-out framing with an order slip for the ultrasound. Patients in the Letter + Order + Incentive arm were also sent a letter and order slip in addition to a $20 unconditional incentive.

All research was conducted in accordance with both the Declarations of Helsinki and Istanbul. The study was approved by the Institutional Review Board at the University of Pennsylvania. A waiver of informed consent was obtained because the study was minimal risk to patients and could not have been practicably carried out without the waiver.^27^ The protocol and statistical plan appear as a Supplement, http://links.lww.com/HC9/A704, and the protocol was registered at ClinicalTrials.gov (NCT04248816). All authors had access to the study data, and reviewed and approved the final manuscript.

### Study population

From our electronic health record (EHR), we identified all patients who were 18 years or older with a current diagnosis of cirrhosis or advanced fibrosis (as defined by the clinician in the EHR), were followed by gastroenterology or hepatology (with one or more visit to any gastroenterology or hepatology practice in the preceding 2 years) and who lived in the Philadelphia Metropolitan Statistical Area. For initial automated extraction, we used International Classification of Disease (ICD-10) codes (K74.60, K74.69, K70.31, K70.30) or cirrhosis in the active problem list of the EHR or in encounter diagnoses in the previous 3 years. Patients were excluded if they had completed liver cancer surveillance within the past 7 months, had a surveillance imaging test scheduled, or a different imaging modality (MRI or CT) recommended by their physician. Patients were also excluded if they had a history of HCC or other liver cancer diagnosis, history of liver transplant, metastatic cancer, or were receiving hospice care. Similarly, ICD-10 codes and the problem list were used for automated data extraction, complemented by manual chart review.

Once patients were confirmed as eligible through a manual medical record review, the gastroenterology/hepatology providers were sent a message with a list of their eligible patients through the EHR and given the opportunity to opt their patients out of participation. If a provider chose to opt-out a patient, the patient was excluded from randomization.

### Interventions

Eligible patients were identified and randomized through a series of 3 batches each approximately 3 months apart in September 2020, January 2021, and April 2021. Patients who were eligible and were not opted out of the intervention by their provider were randomized in a 1:2:2 allocation ratio using a computer-generated randomization algorithm stratified by batch. Patients in the Usual care arm received standard of care and were not mailed any study-related outreach. Patients randomized to the Letter + Order arm received a mailed letter describing the importance of HCC surveillance for patients with cirrhosis or advanced fibrosis and with messaging that encouraged them to get a surveillance ultrasound using the included order slip that had already been placed for them (opt-out framing). Patients in the Letter + Order + Incentive arm received the same letter and order slip in addition to a $20 unconditional incentive in the form of a gift card (ClinCard). Abdominal ultrasound orders were placed by a physician member of the research team (Shivani Kastuar), and the patient’s gastroenterology/hepatology provider was listed as the authorizing provider who would receive the results. The orders were then printed by the study team and matched to the personalized letters for mailing. All completed ultrasound results were routed to the patient’s gastroenterologist/hepatologist for follow-up, as per routine practice. At the time of this trial, there were no concurrent and systematic efforts to remind patients who were overdue for HCC surveillance.

Patients who did not complete an abdominal ultrasound or other type of imaging within 2 months from the date of the initial outreach received a reminder letter with similar messaging to the original letter and including the ultrasound order slip. A reminder was not sent if the patient had a future ultrasound scheduled or if a different imaging modality (MRI or CT) was newly recommended by their provider. The investigators were blinded to patient data and randomization, but the research staff were not blinded as they were administering the interventions.

### Study outcomes

The primary outcome was the proportion of patients who completed an abdominal ultrasound within 6 months of initial outreach. The secondary outcome was the proportion of patients who had any HCC imaging within 6 months of initial outreach, including MRI or CT with contrast. Additional outcomes included evaluating differences in the completion rate by age, sex, race/ethnicity, income at the level of zip code, etiology of cirrhosis, provider and specialty, number of gastrointestinal clinic visits, patient portal status (active vs. inactive), and scheduling modality (self-schedule by means of patient portal vs. phone call). Additionally, we evaluated the percentage of imaging exams that were abnormal, resulted in follow-up imaging, resulted in a diagnosis of HCC and follow-up care, and incidental findings during imaging. Data were obtained from the EHR through automated data extraction and chart review for verification.

### Statistical analysis

We estimated a 10% base response rate for the usual care arm based on prior studies and outreach programs. Based on a preliminary data review, we estimated that we would be able to identify approximately 600 eligible patients across the 3 batches. This sample size would provide 80% power to detect a 13 percentage point increase in response rate for the Letter + Order arm compared to the Usual Care arm (estimated 23%) and a 13 percentage point increase for the Letter + Order + Incentive arm compared to the Letter + Order arm (estimated 36%) using a two-tailed chi-squared test of proportions and a type 1 error rate of 0.025, accounting for 2 pairwise comparisons with Bonferroni correction (0.05/2).

The chi-squared test of proportions was used to calculate the differences, 95% CIs, and *p*-values for the main outcome comparisons. Demographic data were obtained from the EHR through automated data extraction. Race/ethnicity was based on self-reported data from the EHR. Median household income was estimated using the American Community Survey 2014–2018 5-year estimates data for median income by the zip code of residence. Imaging appointment and result data were obtained through automated data extraction and validated through additional manual medical record review.

We performed prespecified exploratory subgroup analyses for the primary outcome by age, sex, race/ethnicity, income, etiology of cirrhosis, provider and specialty, prior gastrointestinal clinic visits, and patient portal status. ORs for the treatment effects within subgroups were calculated using a multivariable logistic regression model with interaction terms. The treatment-by-subgroup interaction terms were analyzed one at a time in separate multivariable models. For the purposes of these analyses, age and income were represented as categorical variables. All analyses were performed using Stata version 15.0 (Stata Corp LP, College Station, Texas).

### Qualitative analysis

A random subsample of 135 patients from the 2 intervention arms were called to complete a postintervention phone interview at least 6 months after initial outreach was mailed. A group of patients were randomly selected from each of the three batches proportionate to the batch size. The patients were asked about their experience with and perception of the impact of HCC surveillance outreach. We conducted a thematic analysis of the postintervention patient interviews to explore patient experience with the intervention and screening process.

## RESULTS

### Participants

**FIGURE 1:**
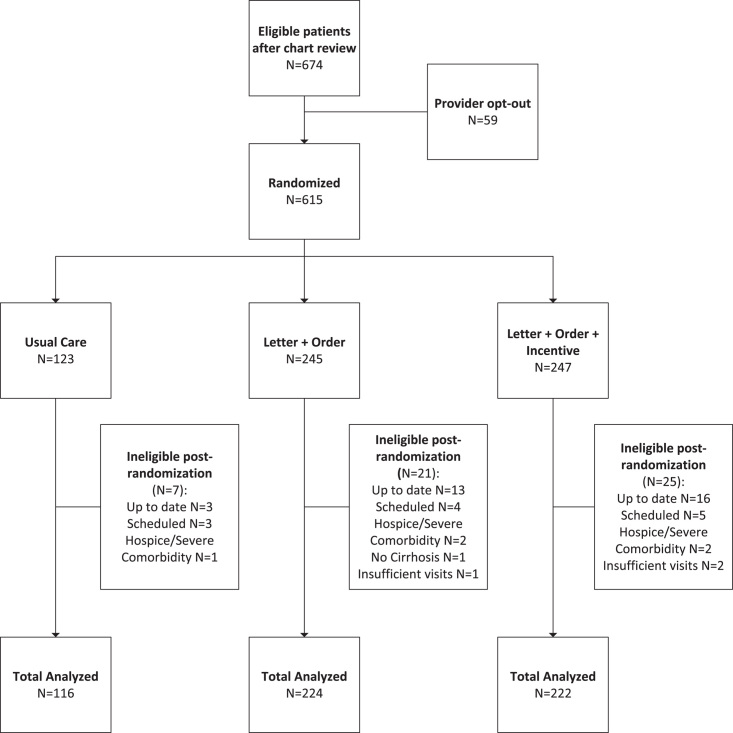
CONSORT flow diagram.

A total of 615 patients were randomized; 53 patients were excluded from analysis postrandomization due to ineligibility (32 were up-to-date on screening, 12 had a future ultrasound scheduled, 5 were on hospice or had a severe comorbidity, 1 did not have a definitive diagnosis of cirrhosis or advanced fibrosis, and 3 had not had at least one visit in the prior 2 years). The proportion of patients excluded postrandomization was evenly distributed across the study arms (Supplemental Table S1, http://links.lww.com/HC9/A705). This resulted in 562 patients included in the final analysis (Figure 1). The mean age was 62.1 (SD 11.1); 56.8% were male; 51.1% were insured by Medicare and 26.3% had commercial insurance; 50.9% were White, 40.6% were Black, and 4.6% were Hispanic or Latino; 69.9% were active electronic patient portal users, and 30.1% had one or more gastroenterology/hepatology clinic visits during the study window (Table 1). The intervention was conducted in three batches: batch 1 from December 1, 2020, through June 1, 2021; batch 2 from February 9, 2021, through August 9, 2021; and batch 3 from May 26, 2021, through November 26, 2021. A total of 280 patients required a reminder message 2 months after the initial outreach: 140 patients in the Letter + Order arm and 140 patients in the Letter + Order + Incentive arm. Each patient was followed for 6 months.

**TABLE 1:** Demographic characteristics

	Usual care (N=116)	Letter + Order (N=224)	Letter + Order + Incentive (N=222)	Total (N=562)
Age				
Mean, SD	61.7 (11.6)	61.4 (10.9)	62.9 (11.0)	62.1 (11.1)
Median, IQR	63 (56.5–69)	63 (55.5–68.5)	64 (56–70)	64 (56–69)
Sex, N (%),				
Male	66 (56.9)	123 (54.9)	130 (58.6)	319 (56.8)
Female	50 (43.1)	101 (45.1)	92 (41.4)	243 (43.2)
Race, N (%)				
White	66 (56.9)	116 (51.8)	104 (46.9)	286 (50.9)
Black or African American	46 (39.7)	88 (39.3)	94 (42.3)	228 (40.6)
Asian	3 (2.6)	9 (4.0)	15 (6.7)	27 (4.8)
Other	1 (0.9)	6 (2.7)	5 (2.3)	12 (2.1)
Unknown or not reported	0	5 (2.2)	4 (1.8)	9 (1.6)
Ethnicity, N (%)				
Not Hispanic or Latino	106 (91.4)	213 (95.1)	207 (93.2)	526 (93.6)
Hispanic or Latino	8 (6.9)	7 (3.1)	11 (5.0)	26 (4.6)
Unknown or not reported	2 (1.7)	4 (1.8)	4 (1.8)	10 (1.8)
Etiology of cirrhosis^a^, N (%)				
Hepatitis C	49 (42.2)	101 (45.1)	103 (46.4)	253 (45.0)
Alcohol-associated liver disease	33 (28.5)	53 (23.7)	63 (28.4)	149 (26.5)
Metabolic dysfunction–associated steatotic liver disease	24 (20.7)	48 (21.4)	36 (16.2)	108 (19.2)
Hepatitis B	8 (6.9)	11 (4.9)	18 (8.1)	37 (6.6)
Autoimmune hepatitis	7 (6.0)	14 (6.3)	11 (5.0)	32 (5.7)
Cryptogenic	4 (3.5)	4 (1.8)	5 (2.3)	13 (2.3)
Other^b^	7 (6.0)	17 (7.6)	14 (6.3)	38 (6.8)
Unspecified	4 (3.5)	12 (5.4)	10 (4.5)	26 (4.6)
Median Household Income by Zip code,^c^ median (IQR)	$55,660.50 ($38,871–$82,883.50)	$56,710 ($34,844–$83,970)	$53,562.50 ($33,786–$84,390)	$55,110.50 ($34,844–$84,134)
Insurance type, N %				
Commercial	32 (27.6)	64 (28.6)	52 (23.4)	148 (26.3)
Medicare	59 (50.9)	108 (48.2)	120 (54.1)	287 (51.1)
Medicaid	24 (20.7)	49 (21.9)	49 (22.1)	122 (21.7)
* *Other	1 (0.9)	3 (1.3)	1 (0.5)	5 (0.9)
Patient portal status, N (%)				
Active	79 (68.1)	160 (71.4)	154 (69.4)	393 (69.9)
Inactive	37 (31.9)	64 (28.6)	68 (30.6)	169 (30.1)
GI clinic visits,^d^ N (%)				
None	87 (75.0)	161 (71.9)	145 (65.3)	393 (69.9)
1 or more	29 (25.0)	63 (28.1)	77 (34.7)	169 (30.1)

a Values do not add up to total because some patients had more than one etiology of cirrhosis.

b Other includes other, primary biliary cirrhosis, primary sclerosing cholangitis, hemochromatosis, and Budd-Chiari syndrome.

c American Community Survey (2014–2018) Median Household Income in 2018 inflated dollars.

d Number of gastroenterology clinic visits during the study window.

Abbreviations: IQR, interquartile range; GI, gastrointestinal.

### Ultrasound completion

Six months after the initial outreach, 27.6% (95% CI: 19.5–35.7) completed an abdominal ultrasound in the Usual care arm, 54.5% (95% CI: 47.9–61.0) completed an abdominal ultrasound in the Letter + Order arm, and 54.1% (95% CI: 47.5–60.6) completed an abdominal ultrasound in the Letter + Order + Incentive arm. There was a significant increase in the ultrasound response rate in the Letter + Order arm compared to the Usual care arm (absolute difference of 26.9%; 95% CI: 16.5–37.3; *p*<0.001), but no significant increase in response rate in the Letter + Order + Incentive arm compared to the Letter + Order arm (absolute difference of −0.4; 95% CI: −9.7 to 8.8; *p*=0.93) (Table 2). There were similar results when including all patients randomized (Supplemental Table S2, http://links.lww.com/HC9/A705).

**TABLE 2:** Proportion of patients completing abdominal ultrasound in the 6-month period

		Usual care vs. Letter + Order		Letter + Order vs. Letter + Order + Incentive	
	Completion N (%; 95% CI)	Difference % (95% CI)	*p* ^a^	Difference % (95% CI)	*p* ^a^
Usual Care (N=116)	32 (27.6; 19.5–35.7)	—	—	—	—
Letter + Order (N=224)	122 (54.5; 47.9–61.0)	26.9 (16.5–37.3)	<0.001	—	—
Letter + Order + Incentive (N=222)	120 (54.1; 47.5–60.6)	—	—	−0.4 (−9.7 to 8.8)	0.93

a *p*-value of <0.025 was the threshold for statistical significance using the Bonferroni correction for multiple comparisons (0.05/2).

### Any imaging completion

At 6 months after the initial outreach, 59.4% (95% CI: 52.9–65.8) completed any imaging (US, MRI, or CT) in the Letter + Order arm, 57.2% (95% CI: 50.7–63.7) completed any imaging in the Letter + Order + Incentive arm, and 32.8% (95% CI: 24.2–41.3) completed any imaging in the Usual Care arm. There was a significant increase in the imaging response rate in the Letter + Order arm compared to the Usual care arm (absolute difference of 26.6%; 95% CI: 15.9–37.3; *p*<0.001), but no significant increase in response rate in the Letter + Order + Incentive arm compared to the Letter + Order arm (absolute difference of −2.2; 95% CI: −11.3 to 7.0; *p*=0.64) (Table 3).

**TABLE 3:** Proportion of patients completing any imaging (US, MRI, or CT) in the 6-month period

		Usual Care vs Letter + Order		Letter + Order vs Letter + Order + Incentive	
	Completion N (%; 95% CI)	Difference % (95% CI)	*p* ^a^	Difference % (95% CI)	*p* ^a^
Usual Care (N=116)	38 (32.8; 24.2–41.3)	—	—	—	—
Letter + Order (N=224)	133 (59.4; 52.9–65.8)	26.6 (15.9–37.3)	<0.001	—	—
Letter + Order + Incentive (N=222)	127 (57.2; 50.7–63.7)	—	—	−2.2 (−11.3 to 7.0)	0.64

a *p*-value of <0.025 was the threshold for statistical significance using the Bonferroni correction for multiple comparisons (0.05/2).

### Screening outcomes

A total of 298 patients across arms completed any imaging (US, MRI, or CT) in the 6-month period following the initial outreach. Of those, 14 (4.7%) had abnormal results or required additional follow-up evaluation. Based on medical record review to date, 11 (78.6%) were ordered follow-up imaging, 9 (81.8%) completed follow-up imaging, and 4 (44.4%) were diagnosed with HCC (Table 4). The Barcelona Clinic Liver Cancer staging among those diagnosed with HCC was as follows: one stage A, two stage B, and one stage C. All 4 patients diagnosed with HCC were in the intervention groups and were referred for treatment. No cases of cancer were identified in the Usual care group.

**TABLE 4:** Patients with any abnormal liver imaging (US, MRI, or CT) and subsequent care

	Usual Care, n/N (%)	Letter + Order, n/N (%)	Letter + Order + Incentive, n/N (%)	Total, n/N (%)
Abnormal	0/38 (0.0)	5/133 (3.8)	9/127 (6.3)	14/298 (4.7)
Follow-up ordered	—	4/5 (80.0)	7/9 (77.8)	11/14 (78.6)
Follow-up completed	—	3/4 (75.0)	6/7 (85.7)	9/11 (81.8)
Diagnosed with HCC	—	0/3 (0.0)	4/6 (66.7)^a^	4/9 (44.4)^a^
Incidental findings	7/38 (18.4)	11/133 (8.3)	16/127 (12.6)	34/298 (11.4)

a Barcelona Clinic Liver Cancer (BCLC) stage at diagnosis: 1 stage A, 2 stage B, and 1 stage C.

Among the 298 patients who completed imaging, 34 (11.4%) had incidental findings that were not liver related (Table 4).

### Subgroup analyses

The subgroup analysis demonstrated a similar response to the interventions across demographic and clinical factors, with no significant interaction terms when accounting for multiple comparisons (Supplemental Figure S1, http://links.lww.com/HC9/A706, Supplemental Figure S2, http://links.lww.com/HC9/A707). There may have been higher response to opt-out outreach among female patients, Black patients, those with no gastrointestinal clinic visits in the prior year, and those with an inactive patient portal.

### Qualitative interviews

A total of 135 patients in the intervention arms (opt-out and incentive arms only) were randomly selected to be contacted for a postintervention interview (stratified by batch). Of these, 18 interviews (13.3%) were completed, 37 declined (27.4%), 69 were unreachable (51.1%), and 11 had other issues (8.1%). Of the 5 that specifically remembered the letter, 4 found it to be useful. Almost half of the patients (n=7) preferred to schedule the ultrasound by phone, while one third (n=6) preferred to schedule during the office visit. Five patients found the patient portal useful for engagement, and 4 patients had issues with receiving mail from the postal service due to reliability. Five patients had to put off liver surveillance due to the COVID-19 pandemic, 3 patients appreciated the gift card, and 3 had issues with transportation to receive the ultrasound.

## DISCUSSION

In this study, we found that mailed outreach with opt-out framing and a signed preorder doubles the participation in HCC surveillance. However, there was no additional effect due to the $20 unconditional financial incentive when added to the mailed outreach intervention. Importantly, we did not find differential effectiveness by sociodemographic characteristics in the subgroup analyses. In the exploratory analysis, we also found that more cancers were identified in the intervention group than in the control group.

The effectiveness of the opt-out intervention may be explained by a few factors. First, it provided direct mail to remind patients about surveillance in a population health approach that does not rely on a visit to a specialist physician. Many patients may not be scheduled or may not attend a visit, which is the conventional opportunity for clinicians to remind patients about surveillance. The subgroup analysis showed that there may have been a greater effect among those without a visit during the trial period. This intervention provided an opportunity to participate during the COVID-19 pandemic, when patients may have missed routine visits. Additionally, the letter served as a nudge to remind patients if the ultrasound was ordered many months before but was forgotten. The letter also used preordering with opt-out framing, implying that participation in HCC surveillance is the default and the patient would have to actively choose not to participate. This has been shown to increase screening for colorectal cancer and hepatitis C in other trials.^20,28^ The preorder also minimized effort for the patient to obtain an order slip and, as the orders were placed by a physician member of the study team, also reduced effort for the clinicians who would typically have to sign each individual order. Prior studies have shown that a lack of an appointment with a specialist and a lack of an order for imaging were barriers to receipt of surveillance.^9,29^

This study supports and adds to the literature on direct outreach for HCC surveillance. A trial at a safety net health system across all practices (not just specialty) evaluating mailed outreach with telephone follow-up showed a similar increase in one-time surveillance from 24.3% to 44.5%, with continued effectiveness over 18 months and a modest benefit from navigation.^30,31^ A follow-up multicenter study across 3 health systems showed an increase in surveillance from 21.9% to 35.1%.^32^ Our study was conducted solely in a gastroenterology and hepatology clinical practice and did not have any telephone follow-up, although there was a mailed reminder. From a scalable standpoint, this simplified the outreach process and required less effort, making it easier to implement in routine practice without requiring significant effort to make phone calls. Our study included a printed ultrasound order slip with mailing to provide opt-out framing and endowment (a sense of ownership by the patient), and to minimize effort for the gastroenterology/hepatology clinician in signing the individual orders.

There are a few reasons why the financial incentive may not have increased response rate. First, the $20 incentive may not have been large enough to overcome any barriers for the patients to attend the ultrasound. Larger incentives of $100 have been shown to increase participation in screening colonoscopy among health system employees. The unconditional incentive may not have had as much effectiveness as other behaviorally informed incentives in this context, such as conditional or lottery-based incentives,^26^ although in a mailed colorectal cancer screening outreach study, unconditional had the highest response rate, despite no effect from any incentive.^21^ There also may be other barriers to surveillance participation that could not be overcome by incentives such as fear of the results or lack of knowledge or belief in surveillance for this population. A survey of patients with cirrhosis showed that a third of patients did not believe that surveillance was necessary without symptoms or an abnormal physical exam, and many patients cited difficulty scheduling, cost, and transportation issues as barriers to surveillance.^33^ As this financial incentive was paired with mailed outreach with preordering, there may not have been any additional patients who would benefit that did not already respond to the other intervention. Finally, financial incentives may not be effective altogether in this patient population.

The main strength of this study was the prospective randomization that controlled for unobserved variables and allowed for the evaluation of the effect of each specific intervention. It was conducted in a pragmatic design in partnership with routine clinical operations; therefore, the results can be generalized to other practices. We also evaluated a scalable approach to surveillance that can leverage EHR processes such as preordering and letter generation. A future state may be able to leverage a bulk ordering process that has been used in other preventive health exams. The study population had a high percentage of Black and Medicaid patients, groups who typically have known disparities and worse outcomes for liver disease.

This study has some important limitations. While we showed the benefit of one-time surveillance, repeat surveillance over time is required to effectively reduce the burden of HCC, although prior studies have shown that this process can be repeated and sustained. This was conducted at an academic tertiary care center, so it may not translate to other care settings. Surveillance performed outside of the health system may have been missed. Although we conducted a chart review and allowed clinicians to opt-out of ineligible patients, we may have missed exams in the outcome ascertainment, and this could have biased toward higher effectiveness in the intervention arms. Additionally, this trial was conducted during the COVID-19 pandemic, although clinical visits and imaging appointments were available at that time.

In summary, we found that sending mailed outreach with orders increased the response to HCC surveillance in a diverse population of patients followed by gastroenterology/hepatology specialists. This approach can be used by health systems to increase adherence to guidelines for population health management in patients with advanced liver disease. Additional research is needed to evaluate its long-term effectiveness in different populations.

## Supplementary Material

SUPPLEMENTARY MATERIAL
