# Supplementary material for: A randomized trial of mailed outreach with behavioral economic interventions to improve liver cancer surveillance
Source: Hepatol Commun. 2023 Dec 15;8(1):e0349. doi: 10.1097/HC9.0000000000000349 (PMC10727671; doi:10.1097/HC9.0000000000000349)
Supplement: SUPPLEMENTARY MATERIAL [file hc9-8-e0349-s001.pdf]

Supplement

Increasing Surveillance Rates for Hepatocellular Carcinoma among Cirrhotic Patients

This supplement provides additional information about the work. It contains the following items:

Initial Protocol ……………………………………………………………………1

Final Protocol ……………………………………………………………………11

Summary of protocol changes ……………………………………………………22

Original statistical analysis plan …………………………………………………..25

Final statistical analysis plan ……………………………………………………..26

Summary of statistical analysis plan changes ……………………………………27

Appendix A: Mailed Letter Templates ……………………………………………28

Appendix B: Post-Intervention Survey ……………………………………………32

Initial Protocol

Increasing surveillance rates for hepatocellular carcinoma among cirrhotic patients

Abstract

This is a 3-arm pilot randomized controlled trial applying behavioral economic approaches (opt-out framing and financial incentives) to encourage patients with liver cirrhosis to complete regular surveillance ultrasounds which may allow for earlier diagnosis of and better outcomes for hepatocellular carcinoma (HCC).

Study Instruments

A sub-sample of 200 patients (100 from each intervention arm) will be called to complete a questionnaire over the phone 6 months after initial outreach was mailed. The subjects will confirm their eligibility (e.g. that they had received outreach about HCC surveillance) and be asked about their experience with and perception of the impact of HCC surveillance outreach.

Group Modifications

Subjects in the usual care arm will not receive a post-outreach phone questionnaire since these subjects will not be sent outreach materials.

Method for Assigning Subjects to Groups

Subjects will be randomly assigned Study ID numbers and then randomized to one of three study arms in a 1:2:2 ratio using a computer-generated randomization algorithm. The randomization will occur in two batches 3 months apart and will be stratified by batch. The research coordinator will record the randomization assignments on a master list which will be maintained on a password protected computer. The research staff will assemble the mailings based on this master list.

Administration of Surveys and/or Process

200 subjects will be randomly selected for the follow-up interview. We anticipate the post outreach interview to take 5-10 minutes to complete over the phone. The research staff will make no more than three attempts to speak directly with the subject. Based on a previous project where we reached about 50% of patients via phone call, we anticipate reaching approximately 100 subjects (50 in each arm) to complete this sub-sample interview.

Data Management

Information about study subjects will be kept confidential and managed according to the requirements of the Health Insurance Portability and Accountability Act of 1996 (HIPAA). Source documents are maintained in PennChart. No source documents will be printed or maintained in paper form at the study site. Data from PennChart will be recorded in Penn Medicine's REDCap system. The investigator and study team will have access to PHI within PennChart and REDCap. We will label all PHI within REDCap as identifiable information so that de-identified exports are possible. All reports that include identifiable information will be stored on the Innovation Center secure drive, maintained behind the UPHS firewall. Direct identifiers will be maintained on RedCap until manuscript publication in case additional chart review is needed for confirmation of results. Once data analysis and manuscripts have been published, direct identifiers will be deleted from RedCap and the de-identified database will be stored on the Innovation Center secure drive.

Objectives

1.1 Objectives

Aim 1: To evaluate if a proactive approach to facilitated HCC surveillance outreach that incorporates opt-out framing increases participation as compared to usual care.

Aim 2: To evaluate if an unconditional incentive informed by behavioral economics increases response to facilitated HCC surveillance outreach.

1.2 Primary outcome variable(s)

The primary outcome is the proportion of subjects who have a surveillance abdominal ultrasound in the six-month period after the study begins.

1.3 Secondary outcome variable(s)

The secondary outcome is the proportion of subjects who have any hepatocellular carcinoma surveillance in the six-month period after the study begins.

Additional variables include the etiology of cirrhosis, demographic and socioeconomic characteristics of subjects who participate, number of clinic visits, as well as exploratory qualitative data regarding experience with outreach. Additionally, we will track the percentage of HCC surveillance images that are abnormal, result in follow-up imaging, and result in a diagnosis of HCC and follow-up care.

Background

There is a substantial burden of HCC-related morbidity and mortality: The age-adjusted incidence rates of HCC have tripled in the US since the 1980s due to the burden of hepatitis C virus (HCV) and the epidemic of non-alcoholic fatty liver disease (NAFLD). The overwhelming majority of HCC in the US occurs in the setting of cirrhosis. The age group most affected by cirrhosis and HCC are baby boomers given that HCV is the leading risk factor for cirrhosis and HCC, followed by NAFLD, hepatitis B virus, and alcohol. The current burden of HCC translates to more than 30,000 new HCC diagnoses every year, with greater than 20,000 HCC-related deaths annually. The incidence of HCC is projected to increase over the next 10-20 years.

Early diagnosis of HCC dictates survival: The American Association for the Study of Liver Diseases (AASLD) recommends biannual HCC surveillance for all patients with cirrhosis using an abdominal ultrasound. These guidelines seek to maximize early diagnosis of HCC which leads to earlier detection and improved survival because early-stage HCC is curable, with 70% 5-year survival compared to 5% in advanced disease.

HCC surveillance rates are suboptimal: Despite longstanding published guidelines for HCC surveillance, adherence is low, with surveillance rates ranging from 15-30% in the US. Two RCTs have tested interventions to increase HCC surveillance, including electronic reminders for primary care providers and mailed reminders (with or without navigators), but neither has been scalable, produced durable responses, or increased surveillance rates above 50%.

HCC surveillance rates at HUP are low: Several thousand patients with advanced liver disease (cirrhosis) receive medical care at the University of Pennsylvania Health System, largely at the outpatient clinic managed by the hepatologists within the Division of Gastroenterology, and the transplant hepatologists through the multi-disciplinary Liver Transplant program. Despite slight differences in the definition of

compliance, the percentage of cirrhotic patients receiving outpatient care at HUP who were compliant with HCC surveillance remains limited.

Statistical Considerations

1.1 Power and sample size

Approximately 700 potentially eligible subjects will be identified in the initial batch via a data abstraction by the Clarity database. Approximately 160 newly eligible patients will be identified in the second batch via a data abstraction by Clarity conducted 3 months after the initial pull. Based on preliminary review, we estimate that 30% of patients complete screening outside of Penn or will be ineligible based on chart review, leaving approximately 600 eligible patients. As such, we anticipate we will have enough patients to enroll at least 600 subjects (and randomize in a 1:2:2 ratio to usual care, opt-out, and incentive arms). We estimate a base return rate for the usual care arm to be 10%. We will consider a meaningful increase in response rate to be 13 percentage points for the opt-out arm as compared to usual care, and 13 percentage points for the incentive arm as compared to the opt-out arm. This will be sufficient sample size using a two-tailed chi-squared test of proportions with 80% power and a Type 1 error rate of .025, accounting for two pairwise comparisons with Bonferroni correction (.05/2 = .025).

1.2 Data analysis

We will conduct a chi-square test of proportions analysis using Stata to compare arm 2 to arm 1 and arm 3 to arm 2 separately using intent-to-treat protocol for the ultrasound completion and any HCC surveillance imaging completion. As exploratory analyses, we will evaluate response by age, gender, race/ethnicity, income at the level of zip code, and etiology of cirrhosis. Analysis will be conducted at least six months after initial outreach.

Study Design

1.1 Design

Randomized: This is a 3-arm randomized controlled trial. Patients with cirrhosis will be identified from eligible patients at the Penn Gastroenterology clinic via medical records query. Their providers will be contacted with the opportunity to opt-out on behalf of the patients. Approximately 490 eligible patients identified in the initial batch will be randomized 1:2:2 into a usual care arm and 2 intervention arms using a computer-generated randomization algorithm (Figure 1). Three months after the initial batch, approximately 110 newly eligible patients will be identified and randomized as described above (Figure 1). Randomization will be stratified by batch. The research coordinator will record the randomization assignments on a master list which will be maintained on a password protected computer. The usual care arm (120 patients) will receive standard of care. The first intervention arm (240 patients) will involve facilitated outreach and opt-out framing, and research staff will send a letter to those patients encouraging them to get a surveillance ultrasound and include an order slip for them to get it done at a health system facility. The second intervention arm (240 patients) will be the same process but will also offer an unconditional incentive of $20 compensation.

Blinding: The investigators will be blinded to the randomization assignment. The research staff will be unblinded. The blinding may be broken for clinical care purposes.

Figure 1. Batch Accrual and Randomization Diagram

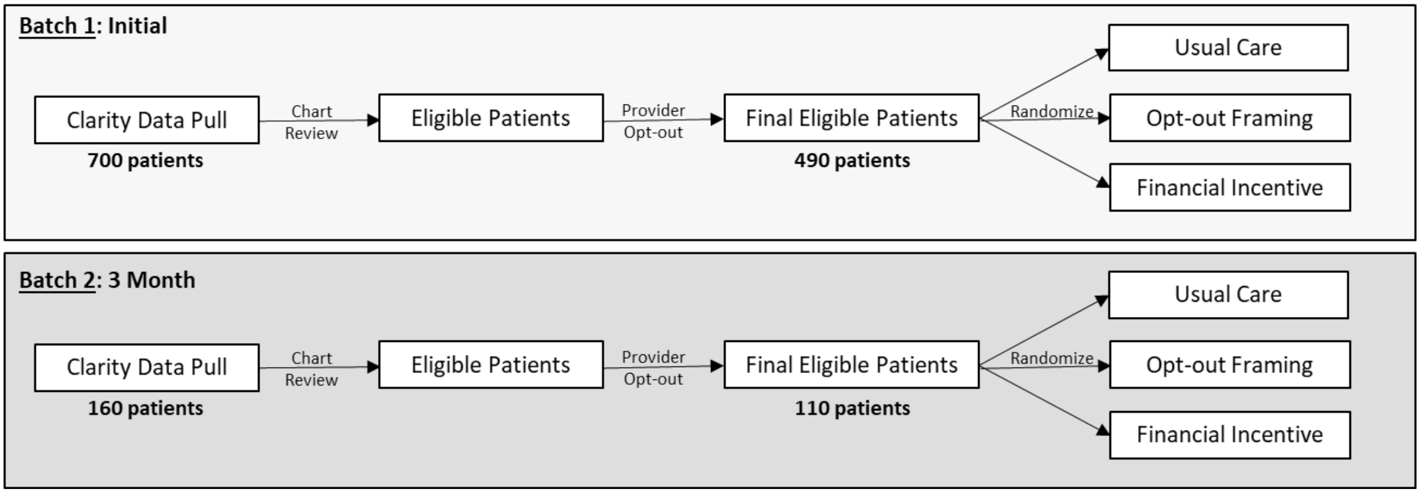

Study duration

We anticipate conducting chart review for two months, mailed outreach and reminder follow-up for two months, waiting for completion of screening for an additional four months, conducting the sub-sample survey for one month, and data analysis and manuscript compilation for 3 months. Thus, we anticipate this project to last 12 months. We anticipate screening ultrasounds to take approximately 1 hour. For the subsample of participants completing the follow-up survey, we would expect the phone call to take approximately 15 minutes.

Resources necessary for human research protection

Dr. Mehta and Dr. Rothstein along with Project Manager Catherine Reitz and Clinical Research Coordinator Caitlin McDonald are adequately informed of the protocol and adequately qualified to conduct research via training required for medical doctors/students and research coordinators. All are up to date with HIPAA and CITI training.

Characteristics of the Study Population

Target population

The study population includes patients with cirrhosis who receive care at any Penn Gastroenterology clinic and are overdue for HCC surveillance.

Subjects enrolled by Penn Researchers

600

Subjects enrolled by Collaborating Researchers

0

Accrual

Through automated data extraction from Clarity, we will identify potentially eligible patients. The research team will review the electronic medical record charts in EPIC to confirm study eligibility. Providers of eligible patients will be sent an email that allows them to opt out of participation on behalf of their patients. Patients who are eligible and whose providers do not opt out of the intervention will be randomized into one of the three arms of the intervention.

Based on preliminary review of the data, we anticipate approximately 860 screening eligible patients: 700 in the initial batch and 160 in the second batch. We know that roughly 30% of patients complete screening outside of Penn or will be ineligible based on chart review, leaving approximately 600 eligible patients.

Key inclusion criteria

Patients who are 18+ years old with a current diagnosis of cirrhosis receiving care at any Penn Gastroenterology/Hepatology practice, who must have had 2 or more visits to a Penn Gastroenterology/Hepatology practice in the preceding two years, and who must live in the Philadelphia Metropolitan Statistical Area. All patients meeting these criteria will be included regardless of race, ethnicity, or gender.

Key exclusion criteria

Patients with a history of HCC diagnosis and/or have completed screening within the past 9 months. We will also exclude patients with metastatic cancer or receiving hospice care.

Vulnerable Populations
No vulnerable populations are included in the research study.

Populations Vulnerable to Undue Influence or Coercion
We are not specifically targeting any vulnerable populations.

Subject Recruitment

Through automated data extraction from Clarity, we will identify potentially eligible patients. The research team will review the electronic medical record charts in EPIC to confirm study eligibility. Providers of eligible patients will be sent an EPIC inbox message that allows them to opt out of participation on behalf of their patients. Patients who are eligible and whose providers do not opt out of the intervention will be randomized into one of the three arms of the intervention. We will obtain a waiver of consent for this low-risk intervention as it would not be possible to assess response if we had to obtain consent prior to outreach.

Subject Compensation
Yes, subjects will be financially compensated for their participation.

One intervention arm will receive monetary compensation of a ClinCard worth $20. The other two study arms will not receive compensation.

Procedures

Screening – Once patients are confirmed as eligible, a list will be sent to each provider to opt-out of the study on the patient's behalf.

Randomization - Subjects will be randomly assigned Study ID numbers and then randomized in two batches in a 1:2:2 ratio to one of three arms using a computer-generated randomization algorithm stratified by batch. The research coordinator will record the randomization assignments on a master list which will be maintained by the research coordinator on a password protected computer. The research staff will assemble the mailings based on this master list.

Intervention - Patients will either receive standard of care (usual care group), receive a letter encouraging them to get a surveillance ultrasound plus an order slip for the procedure (opt-out), or receive the letter and order slip plus an unconditional incentive of $20 (incentive). Patients then have the option to complete the ultrasound. If they have not completed the ultrasound within 2 month from initial outreach, the research staff will send a reminder similar to the original messaging and including the order slip.

Sub-sample Questionnaire - A sub-sample of 200 patients will be called to complete a questionnaire over the phone 6 months after initial outreach was mailed. The subjects will confirm their eligibility (e.g. that they had received outreach about HCC surveillance) and be asked information about their qualitative experience with the outreach materials and approach. We anticipate these questionnaires to take no more than 5-10 minutes to complete over the phone. The research staff will make no more than three attempts to speak directly with the subject. Interviews will be recorded and transcribed.

Analysis Plan

1.1 Power and Sample Size

Approximately 860 potentially eligible subjects will be identified via a data abstraction by the Clarity database. Based on preliminary review, we estimate that 30% of patients complete screening outside of Penn or will not meet eligibility criteria per chart review, leaving approximately 600 eligible patients. As such, we anticipate we will have enough patients to enroll at least 600 subjects (and randomize in a 1:2:2 ratio to usual care, opt-out, and incentive arms). We estimate a base return rate for the usual care arm to be 10%. We will consider a meaningful increase in response rate to be 13 percentage points for the opt-out arm as compared to usual care, and 13 percentage points for the incentive arm as compared to the opt-out arm. This will be sufficient sample size using a two-tailed chi-square test of proportions with 80% power and a Type 1 error rate of .025, accounting for two pairwise comparisons with Bonferroni correction (.05/2 = .025).

1.2 Data analysis

We will conduct a chi-squared test of proportions analysis using Stata to compare arm 2 to arm 1 and arm 3 to arm 2 separately using intent-to-treat protocol for the ultrasound completion and any HCC surveillance imaging completion. As exploratory analyses, we will evaluate response by age, gender, race/ethnicity, income at the level of zip code, and etiology of cirrhosis. Analysis will be conducted at least six months after initial outreach.

Qualitative analysis of the post-intervention phone interviews will be conducted using NVivo. This will include thematic analysis of patient experience with the intervention and screening process.

Analysis will be conducted by blinded members of the research team at least 6 months after the mailings have been sent.

Data Confidentiality

Paper-based records will be kept in a secure location and only be accessible to personnel involved in the study. Computer-based files will only be made available to personnel involved in the study through the use of access privileges and passwords. Wherever feasible, identifiers will be removed from study-related information. Audio and/or video recordings will be transcribed and then destroyed to eliminate audible identification of subjects.

Subject Confidentiality

Information about study subjects will be kept confidential and managed according to the requirements of the Health Insurance Portability and Accountability Act of 1996 (HIPAA). All PHI will be maintained on UPHS servers. Source documents are maintained in PennChart. No source documents will be printed or maintained in paper form at the study site. Data from PennChart will be recorded in Penn Medicine's REDCap system. The investigator and study team (which includes the research coordinator, and research assistants) will have access to PHI within PennChart and REDCap. We will label all PHI within REDCap as identifiable information so that de-identified exports are possible. All reports that include identifiable information will be stored on the Innovation Center secure drive, maintained behind the UPHS firewall. Direct identifiers will be maintained on RedCap until manuscript publication in case additional chart review is needed for confirmation of results. Once data analysis and manuscripts have been published, direct identifiers will be deleted from RedCap and the de-identified database will be stored on the Innovation Center secure drive. Phone calls will be transcribed using Datagain Transcription, a HIPAA compliant transcription service. Datagain's system controls, database architecture and internal policies provide HIPAA compliance.

Sensitive Research Information

This Research does not involve collection of sensitive information about the subjects that should be excluded from the electronic medical record.

Subject Privacy

Because this study involves sending a letter to patients which will include PHI such as name, etc., research staff will be required to check the address listed for the patient a second time after writing out the envelope to ensure it is not sent to the wrong address/wrong person. Research staff will also be required to check that the documents are addressed to the correct person prior to sealing the envelope. We will only interact with the subsample of subjects with which we plan to call to conduct a follow-up questionnaire. With these subjects, we will conduct phone calls in a private area. When we call subjects, we will confirm the identify before administering the questionnaire. We will not be interacting with subjects in person.

Data Disclosure

Completed surveillance ultrasound results will be disclosed to the participant's provider for continuity of care.

Protected Health Information/Data Protection

- Name
- Street address, city, county, precinct, zip code, and equivalent geocodes

- All elements of dates (except year) for dates directly related to an individual and all ages over 89
- Telephone and fax numbers
- Medical record numbers
- Biometric identifiers, incl. finger and voice prints

Consent Process

1.1 Overview

We are requesting a waiver of consent as this intervention is low risk and does not negatively impact typical standard of care procedures for these patients at Penn. The research presents no more than minimal risk of harm to subjects and involves no procedures for which written consent is normally required outside of the research context. Surveillance ultrasound are clinically available and utilized tests for HCC screening. The research related activity is the randomization of subjects to different outreach strategies and providing them with orders to help complete surveillance imaging.

Subjects rights and welfare will not be adversely affected by the waiver of authorization and consent. All subjects will have the opportunity to voluntarily participate in HCC screening. Each arm has the opportunity to engage in HCC screening through routine care as well.

We believe that we would not be able to practicably conduct the research without waiver of consent. If we had to obtain either written or verbal consent ahead of time, it would substantially limit our study population and it may differentially alter their participation in the intervention. Thus, we would only learn about the response rate for patients who we were able to speak to for consent. This would limit the generalizability to practice. Obtaining waiver of consent would allow us to avoid the potential selection/volunteer bias for inclusion of patients particularly interested in screening that can occur when consent is required. Since our main objective is to understand the potential influence varying outreach strategies on subject behavior, we believe that obtaining consent would compromise our primary objective. We have received waiver of consent for similar studies related to cancer screening outreach in the past.

Verbal consent will be obtained from the subsample with whom we plan to conduct post-intervention interviews. A randomly selected sub-sample of 200 patients (100 from each intervention arm) will be called by the research staff to complete a questionnaire over the phone 6 months after initial outreach was mailed. No more than three phone call attempts will be made to reach the patients. The subjects will be informed about the purpose of the phone call, asked if they would like to participate and if the phone call can be recorded. The phone interview will consist of questions about patient experience, how to improve patient experience, and perception of the impact of HCC surveillance outreach (please see attached phone script). Since these interviews will be conducted over the phone, verbal consent will be obtained and recorded in RedCap. All recordings will be de-identified and stored on the Innovation Center secure drive. Once the de-identified recordings have been transcribed, the audio recording will be deleted. The de-identified transcripts will be stored on the Innovation Center secure drive.

1.2 Children and Adolescents

None

1.3 Adult Subjects Not Competent to Give Consent

Waiver of consent is being requested.

Waiver of Consent

1.1 Minimal Risk

The study involves no more than minimal risk to subjects and involves no procedures for which written consent is normally required outside of the research context. Surveillance ultrasound are clinically available and utilized tests used to screen for HCC. The research related activity is the randomization of subjects to different outreach strategies and providing them with orders to help complete surveillance imaging.

1.2 Impact on Subject Rights and Welfare

Subjects rights and welfare will not be adversely affected by the waiver of authorization and consent. All subjects will have the opportunity to voluntarily participate in HCC screening. Each arm has the opportunity to engage in HCC screening through routine care as well.

1.3 Waiver Essential to Research

We believe that we would not be able to practically conduct the research without waiver of consent. If we had to obtain either written or verbal consent ahead of time, it would substantially limit our study population and it may alter their participation in the intervention. Thus, we would only learn about the response rate for patients who we were able to speak to for consent. This, would limit the generalizability to practice. Obtaining waiver of consent would allow us to avoid the potential selection/volunteer bias for inclusion of patients particularly interested in screening that can occur when consent is required. Since our main objective is to understand the potential influence varying outreach strategies on subject behavior, we believe that obtaining consent would compromise our primary objective. We have received waiver of consent for similar studies related to cancer screening outreach in the past.

1.4 Additional Information to Subjects

Subjects will be sent a letter explaining that we are offering a special program to patients overdue for ultrasound. Subjects will receive information about the risks and benefits of surveillance ultrasound for HCC screening. Providers will disclose screening results directly to patients if completed.

Potential Study Risks

The risks associated with this study are no more than minimal. Loss of confidentiality is possible, but unlikely. We will minimize this risk by using de-identified information whenever possible and by maintaining all identifiable information on a secure drive and/or in a HIPAA-compliant system (e.g. REDCap). There is also the risk of psychological harm associated with being screened for cancer. This risk will be minimized by the timely communication of screening test results to the subject and the facilitation of follow up diagnostic testing as needed (as is usual practice for screening outreach programs).

Potential Study Benefits

If a participant completes HCC surveillance screening, which is standard of clinical care, the subjects will potentially benefit from participation by increasing the chances of identifying hepatocellular carcinoma at an early stage. Information from this study may benefit society through a better understanding of how to effectively increase overall participation rates in HCC surveillance according to guidelines.

Data and Safety Monitoring

Safety will be monitored on an ongoing basis by the PI and the study team. The PI or designee will review the study charts to evaluate events at each subject interaction to ensure the grade, relationship to the study procedure, expectedness and the course of action for each subject is documented. We will also participate in a Data Safety and Monitoring Board through NIH and the Penn/CHIBE Roybal pilot program.

Risk / Benefit Assessment

The risks associated with this study are no more than minimal. Better knowledge of how to increase mailed screening could potentially address one of the major barriers of accessing care, i.e. having patients come in for clinical office visits. The Principal Investigator believes that the risks of participating in the study are outweighed by the potential benefits of participating in the study.

Final Protocol

Increasing surveillance rates for hepatocellular carcinoma among cirrhotic patients

****New changes from initial protocol notated in bold, parts removed from initial protocol notated in strikethrough**

Abstract

This is a 3-arm pilot randomized controlled trial applying behavioral economic approaches (opt-out framing and financial incentives) to encourage patients with liver cirrhosis to complete regular surveillance ultrasounds which may allow for earlier diagnosis of and better outcomes for hepatocellular carcinoma (HCC).

Study Instruments

A sub-sample of ~~200~~ ~~**60**~~ **134** patients (~~100~~ ~~**30**~~ **67** from each intervention arm) will be called to complete a questionnaire over the phone 6 months after initial outreach was mailed. The subjects will confirm their eligibility (e.g. that they had received outreach about HCC surveillance) and be asked about their experience with and perception of the impact of HCC surveillance outreach.

Group Modifications

Subjects in the usual care arm will not receive a post-outreach phone questionnaire since these subjects will not be sent outreach materials.

Method for Assigning Subjects to Groups

Subjects will be randomly assigned Study ID numbers and then randomized to one of three study arms in a 1:2:2 ratio using a computer-generated randomization algorithm. The randomization will occur in ~~two~~ **three** batches 3 months apart and will be stratified by batch. The research coordinator will record the randomization assignments on a master list which will be maintained on a password protected computer. The research staff will assemble the mailings based on this master list. **For the post-intervention phone interviews, a proportion of the ~~60~~ 134 subjects will be randomly selected from each batch of randomized participants mailed outreach, based on batch size, using STATA. For example, if batch 1 contained 142 patients out of the total 480 randomized to one of the intervention arms, then 29.6% of the ~~60~~ 134 subjects (equaling approximately ~~18~~ 40) will be randomly selected from batch 1 to be called for the interview.**

Administration of Surveys and/or Process

~~200~~ ~~**60**~~ **134** subjects will be randomly selected for the follow-up interview **with the goal of completing 30 interviews**. We anticipate the post outreach interview to take ~~5-10~~ **approximately 15** minutes to complete over the phone. The research staff will make no more than three attempts to speak directly with the subject. ~~Based on a previous project where we reached about 50% of patients via phone call, we anticipate reaching approximately 100 **30** subjects (50 **15** in each arm) to complete this sub-sample interview.~~ **We originally anticipated reaching about 50% of patients via phone call, however, after contacting the first sample of batch 1 patients we found the response rate to be much lower. Based on this, we have increased our sample from 60 subjects to 134 subjects based on a 25% response rate and taking into account already completed interview outreach.**

Data Management

Information about study subjects will be kept confidential and managed according to the requirements of the Health Insurance Portability and Accountability Act of 1996 (HIPAA). Source documents are maintained in PennChart. No source documents will be printed or maintained in paper form at the study site. Data from PennChart will be recorded in Penn Medicine's REDCap system. The investigator and study team will have access to PHI within PennChart and REDCap. We will label all PHI within REDCap as identifiable information so that de-identified exports are possible. All reports that include identifiable information will be stored on the Innovation Center secure drive, maintained behind the UPHS firewall. Direct identifiers will be maintained on RedCap until manuscript publication in case additional chart review is needed for confirmation of results. Once data analysis and manuscripts have been published, direct identifiers will be deleted from RedCap and the de-identified database will be stored on the Innovation Center secure drive.

Objectives

1.1 Objectives

Aim 1: To evaluate if a proactive approach to facilitated HCC surveillance outreach that incorporates opt-out framing increases participation as compared to usual care.

Aim 2: To evaluate if an unconditional incentive informed by behavioral economics increases response to facilitated HCC surveillance outreach.

1.2 Primary outcome variable(s)

The primary outcome is the proportion of subjects who have a surveillance abdominal ultrasound in the six-month period after the study begins.

1.3 Secondary outcome variable(s)

The secondary outcome is the proportion of subjects who have any hepatocellular carcinoma surveillance in the six-month period after the study begins.

Additional variables include the etiology of cirrhosis, demographic and socioeconomic characteristics of subjects who participate, **differences by provider/specialty,** number of clinic visits, **differences among MyPennMedicine users and non-users,** as well as exploratory qualitative data regarding experience with outreach. Additionally, we will track the percentage of HCC surveillance images that are abnormal, result in follow-up imaging, ~~and~~ result in a diagnosis of HCC and follow-up care**, and incidental findings during imaging**.

Background

There is a substantial burden of HCC-related morbidity and mortality: The age-adjusted incidence rates of HCC have tripled in the US since the 1980s due to the burden of hepatitis C virus (HCV) and the epidemic of non-alcoholic fatty liver disease (NAFLD). The overwhelming majority of HCC in the US occurs in the setting of cirrhosis. The age group most affected by cirrhosis and HCC are baby boomers given that HCV is the leading risk factor for cirrhosis and HCC, followed by NAFLD, hepatitis B virus, and alcohol. The current burden of HCC translates to more than 30,000 new HCC diagnoses every year, with greater than 20,000 HCC-related deaths annually. The incidence of HCC is projected to increase over the next 10-20 years.

Early diagnosis of HCC dictates survival: The American Association for the Study of Liver Diseases (AASLD) recommends biannual HCC surveillance for all patients with cirrhosis using an abdominal ultrasound. These guidelines seek to maximize early diagnosis of HCC which leads to earlier detection and improved survival because early-stage HCC is curable, with 70% 5-year survival compared to 5% in advanced disease.

HCC surveillance rates are suboptimal: Despite longstanding published guidelines for HCC surveillance, adherence is low, with surveillance rates ranging from 15-30% in the US. Two RCTs have tested interventions to increase HCC surveillance, including electronic reminders for primary care providers and mailed reminders (with or without navigators), but neither has been scalable, produced durable responses, or increased surveillance rates above 50%.

HCC surveillance rates at HUP are low: Several thousand patients with advanced liver disease (cirrhosis) receive medical care at the University of Pennsylvania Health System, largely at the outpatient clinic managed by the hepatologists within the Division of Gastroenterology, and the transplant hepatologists through the multi-disciplinary Liver Transplant program. Despite slight differences in the definition of compliance, the percentage of cirrhotic patients receiving outpatient care at HUP who were compliant with HCC surveillance remains limited.

Statistical Considerations

1.1 Power and sample size

Approximately 700 potentially eligible subjects will be identified in the initial batch via a data abstraction by the Clarity database. Approximately 160 newly eligible patients will be identified in the second batch via a data abstraction by Clarity conducted 3 months after the initial pull. Based on preliminary review, we estimate that 30% of patients complete screening outside of Penn or will be ineligible based on chart review, leaving approximately 600 eligible patients. As such, we anticipate we will have enough patients to enroll at least 600 subjects (and randomize in a 1:2:2 ratio to usual care, opt-out, and incentive arms). We estimate a base return rate for the usual care arm to be 10%. We will consider a meaningful increase in response rate to be 13 percentage points for the opt-out arm as compared to usual care **(23%)**, and 13 percentage points for the incentive arm as compared to the opt-out arm **(36%)**. This will be sufficient sample size using a two-tailed chi-squared test of proportions with 80% power and a Type 1 error rate of .025, accounting for two pairwise comparisons with Bonferroni correction (.05/2 = .025).

1.2 Data analysis

We will conduct a chi-square test of proportions analysis using Stata to compare arm 2 to arm 1 and arm 3 to arm 2 separately using intent-to-treat protocol for the ultrasound completion and any HCC surveillance imaging completion. As exploratory analyses, we will evaluate response by age, gender, race/ethnicity, income at the level of zip code, ~~and~~ etiology of cirrhosis**, and provider/specialty. We will also evaluate differences in response rate among MyPennMedicine users and non-users and scheduling modality (MyPennMedicine vs. call). Additionally, we will evaluate the percentage of HCC surveillance images that are abnormal, result in follow-up imaging, result in a diagnosis of HCC and follow-up care, and incidental findings during imaging between the study arms.** Analysis will be conducted at least six months after initial outreach.

Study Design

1.1 Design

Randomized: This is a 3-arm randomized controlled trial. Patients with cirrhosis will be identified from eligible patients at the Penn Gastroenterology clinic via medical records query. Their providers will be

contacted with the opportunity to opt-out on behalf of the patients. Approximately 490 eligible patients identified in the initial batch will be randomized 1:2:2 into a usual care arm and 2 intervention arms using a computer-generated randomization algorithm (Figure 1). Three months after the initial batch, approximately 110 newly eligible patients will be identified and randomized as described above (Figure 1). **Three months after the second batch, a third and final data pull will be completed to identify any remaining patients meeting study criteria.** Randomization will be stratified by batch. The research coordinator will record the randomization assignments on a master list which will be maintained on a password protected computer. The usual care arm (120 patients) will receive standard of care. The first intervention arm (240 patients) will involve facilitated outreach and opt-out framing, and research staff will send a letter to those patients encouraging them to get a surveillance ultrasound and include an order slip for them to get it done at a health system facility. The second intervention arm (240 patients) will be the same process but will also offer an unconditional incentive of $20 compensation.

Blinding: The investigators will be blinded to the randomization assignment. The research staff will be unblinded. The blinding may be broken for clinical care purposes.

Figure 1. Batch Accrual and Randomization Diagram

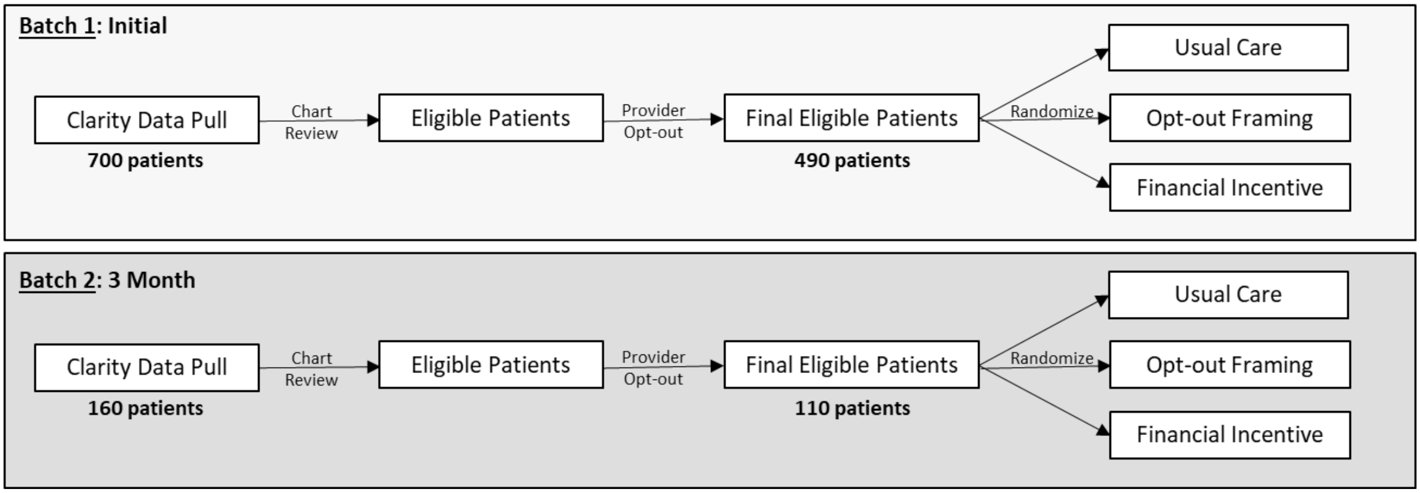

Study duration

We anticipate conducting chart review for two months, mailed outreach and reminder follow-up for two months, waiting for completion of screening for an additional four months, conducting the sub-sample survey for one month, and data analysis and manuscript compilation for 3 months. Thus, we anticipate this project to last 12 months. We anticipate screening ultrasounds to take approximately 1 hour. For the subsample of participants completing the follow-up survey, we would expect the phone call to take approximately 15 minutes.

Resources necessary for human research protection

Dr. Mehta and Dr. Rothstein along with Project Manager Catherine Reitz and Clinical Research Coordinator Caitlin McDonald are adequately informed of the protocol and adequately qualified to conduct research

via training required for medical doctors/students and research coordinators. All are up to date with HIPAA and CITI training.

Characteristics of the Study Population

<u>Target population</u>

The study population includes patients with cirrhosis who receive care at any Penn Gastroenterology clinic and are overdue for HCC surveillance.

<u>Subjects enrolled by Penn Researchers</u>

600

<u>Subjects enrolled by Collaborating Researchers</u>

0

Accrual

Through automated data extraction from Clarity, we will identify potentially eligible patients. The research team will review the electronic medical record charts in EPIC to confirm study eligibility. Providers of eligible patients will be sent an email that allows them to opt out of participation on behalf of their patients. Patients who are eligible and whose providers do not opt out of the intervention will be randomized into one of the three arms of the intervention.

Based on preliminary review of the data, we anticipate approximately 860 screening eligible patients: 700 in the initial batch and 160 in the second batch. We know that roughly 30% of patients complete screening outside of Penn or will be ineligible based on chart review, leaving approximately 600 eligible patients.

Key inclusion criteria

Patients who are 18+ years old with a current diagnosis of cirrhosis **or advanced fibrosis** receiving care at any Penn Gastroenterology/Hepatology practice, who must have had ~~2~~ **1** or more visits to a Penn Gastroenterology/Hepatology practice in the preceding two years **and are currently followed by Penn GI**, and who must live in the Philadelphia Metropolitan Statistical Area. All patients meeting these criteria will be included regardless of race, ethnicity, or gender.

Key exclusion criteria

Patients with a history of HCC **or other liver carcinoma** diagnosis**, history of liver transplant,** and/or have completed screening within the past ~~9~~ **7** months**,** ~~**or**~~ **have a future screening scheduled, or a different screening modality (MRI, CT, etc.) recommended by their physician**. We will also exclude patients with metastatic cancer or receiving hospice care.

Vulnerable Populations
No vulnerable populations are included in the research study.

Populations Vulnerable to Undue Influence or Coercion
We are not specifically targeting any vulnerable populations.

Subject Recruitment

Through automated data extraction from Clarity, we will identify potentially eligible patients. The research team will review the electronic medical record charts in EPIC to confirm study eligibility. Providers of eligible patients will be sent an EPIC inbox message that allows them to opt out of participation on behalf of their patients. Patients who are eligible and whose providers do not opt out of the intervention will be randomized into one of the three arms of the intervention. We will obtain a waiver of consent for this low-risk intervention as it would not be possible to assess response if we had to obtain consent prior to outreach.

Subject Compensation
Yes, subjects will be financially compensated for their participation.

One intervention arm will receive monetary compensation of a **Greenphire** ClinCard worth $20. The other two study arms will not receive compensation.

Procedures

Screening – Once patients are confirmed as eligible, a list will be sent to each provider to opt-out of the study on the patient's behalf.

Randomization - Subjects will be randomly assigned Study ID numbers and then randomized in ~~two~~ **three** batches in a 1:2:2 ratio to one of three arms using a computer-generated randomization algorithm stratified by batch. The research coordinator will record the randomization assignments on a master list which will be maintained by the research coordinator on a password protected computer. The research staff will assemble the mailings based on this master list. **For the post-intervention phone interviews, a proportion of the ~~60~~ 134 subjects will be randomly selected from each batch of randomized participants mailed outreach, based on batch size, using STATA. For example, if batch 1 contained 142 patients out of the total 480 randomized to one of the intervention arms, then 29.6% of the ~~60~~ 134 subjects (equaling approximately ~~18~~ 40) will be randomly selected from batch 1 to be called for the interview.**

Intervention - Patients will either receive standard of care (usual care group), receive a letter encouraging them to get a surveillance ultrasound plus an order slip for the procedure (opt-out), or receive the letter and order slip plus an unconditional incentive of $20 (incentive). Patients then have the option to complete the ultrasound. **A second data pull will be used to determine ultrasound completion at 2 months after initial outreach for all patients in the study.** If they have not completed ~~the ultrasound~~ **screening** within 2 month**s** from initial outreach, the research staff will send a reminder similar to the original messaging and including the order slip. **A reminder will not be sent if the patient has a future ultrasound scheduled or a different screening modality is now recommended. A final data pull will be used to determine ultrasound completion at 6 months from initial outreach for all patients in the study. Some patients may have their screening completed outside of Penn and View-only Care Everywhere encounters that have been downloaded into the patient's chart may be reviewed for this information. Care Everywhere will not be used to request clinical information for research purposes.**

Sub-sample Questionnaire - A sub-sample of ~~200~~ **~~60~~ 134** patients will be called to complete a questionnaire over the phone 6 months after initial outreach was mailed. The subjects will confirm their

eligibility (e.g. that they had received outreach about HCC surveillance) and be asked information about their qualitative experience with the outreach materials and approach. We anticipate these questionnaires to take no more than ~~5-10~~ **15** minutes to complete over the phone. The research staff will make no more than three attempts to speak directly with the subject. Interviews will be recorded and transcribed.

Analysis Plan

<u>1.1 Power and Sample Size</u>

Approximately 860 potentially eligible subjects will be identified via a data abstraction by the Clarity database. Based on preliminary review, we estimate that 30% of patients complete screening outside of Penn or will not meet eligibility criteria per chart review, leaving approximately 600 eligible patients. As such, we anticipate we will have enough patients to enroll at least 600 subjects (and randomize in a 1:2:2 ratio to usual care, opt-out, and incentive arms). We estimate a base return rate for the usual care arm to be 10%. We will consider a meaningful increase in response rate to be 13 percentage points for the opt-out arm as compared to usual care **(23%)**, and 13 percentage points for the incentive arm as compared to the opt-out arm **(36%)**. This will be sufficient sample size using a two-tailed chi-square test of proportions with 80% power and a Type 1 error rate of .025, accounting for two pairwise comparisons with Bonferroni correction (.05/2 = .025).

<u>1.2 Data analysis</u>

We will conduct a chi-squared test of proportions analysis using Stata to compare arm 2 to arm 1 and arm 3 to arm 2 separately using intent-to-treat protocol for the ultrasound completion and any HCC surveillance imaging completion. As exploratory analyses, we will evaluate response by age, gender, race/ethnicity, income at the level of zip code, ~~and~~ etiology of cirrhosis**, and provider/specialty. We will also evaluate differences in response rate among MyPennMedicine users and non-users and scheduling modality (MyPennMedicine vs. call). Additionally, we will evaluate the percentage of HCC surveillance images that are abnormal, result in follow-up imaging, result in a diagnosis of HCC and follow-up care, and incidental findings during imaging between the study arms.** Analysis will be conducted at least six months after initial outreach.

Qualitative analysis of the post-intervention phone interviews will be conducted using NVivo. This will include thematic analysis of patient experience with the intervention and screening process.

Analysis will be conducted by blinded members of the research team at least 6 months after the mailings have been sent.

Data Confidentiality

Paper-based records will be kept in a secure location and only be accessible to personnel involved in the study. Computer-based files will only be made available to personnel involved in the study through the use of access privileges and passwords. Wherever feasible, identifiers will be removed from study-related information. Audio and/or video recordings will be transcribed and then destroyed to eliminate audible identification of subjects.

Subject Confidentiality

Information about study subjects will be kept confidential and managed according to the requirements

of the Health Insurance Portability and Accountability Act of 1996 (HIPAA). All PHI will be maintained on UPHS servers. Source documents are maintained in PennChart. No source documents will be printed or maintained in paper form at the study site. Data from PennChart will be recorded in Penn Medicine's REDCap system. The investigator and study team (which includes the research coordinator, and research assistants) will have access to PHI within PennChart and REDCap. We will label all PHI within REDCap as identifiable information so that de-identified exports are possible. All reports that include identifiable information will be stored on the Innovation Center secure drive, maintained behind the UPHS firewall. Direct identifiers will be maintained on RedCap until manuscript publication in case additional chart review is needed for confirmation of results. Once data analysis and manuscripts have been published, direct identifiers will be deleted from RedCap and the de-identified database will be stored on the Innovation Center secure drive. Phone calls will be transcribed using Datagain Transcription, a HIPAA compliant transcription service. Datagain's system controls, database architecture and internal policies provide HIPAA compliance.

Sensitive Research Information

This Research does not involve collection of sensitive information about the subjects that should be excluded from the electronic medical record.

Subject Privacy

Because this study involves sending a letter to patients which will include PHI such as name, etc., research staff will be required to check the address listed for the patient a second time after writing out the envelope to ensure it is not sent to the wrong address/wrong person. Research staff will also be required to check that the documents are addressed to the correct person prior to sealing the envelope. We will only interact with the subsample of subjects with which we plan to call to conduct a follow-up questionnaire. With these subjects, we will conduct phone calls in a private area. When we call subjects, we will confirm the identify before administering the questionnaire. We will not be interacting with subjects in person.

Data Disclosure

Greenphire ClinCard and the Office of Finance at the University of Pennsylvania will receive participant names, address, and date of birth for subject compensation payments. Completed surveillance ultrasound results will be disclosed to the participant's provider for continuity of care.

Protected Health Information/Data Protection

- Name
- Street address, city, county, precinct, zip code, and equivalent geocodes
- All elements of dates (except year) for dates directly related to an individual and all ages over 89
- Telephone and fax numbers
- Medical record numbers
- Biometric identifiers, incl. finger and voice prints

Consent Process

1.1 Overview

We are requesting a waiver of consent as this intervention is low risk and does not negatively impact typical standard of care procedures for these patients at Penn. The research presents no more than minimal risk of harm to subjects and involves no procedures for which written consent is normally required outside of the research context. Surveillance ultrasound are clinically available and utilized tests for HCC screening. The research related activity is the randomization of subjects to different outreach strategies and providing them with orders to help complete surveillance imaging.

Subjects rights and welfare will not be adversely affected by the waiver of authorization and consent. All subjects will have the opportunity to voluntarily participate in HCC screening. Each arm has the opportunity to engage in HCC screening through routine care as well.

We believe that we would not be able to practicably conduct the research without waiver of consent. If we had to obtain either written or verbal consent ahead of time, it would substantially limit our study population and it may differentially alter their participation in the intervention. Thus, we would only learn about the response rate for patients who we were able to speak to for consent. This would limit the generalizability to practice. Obtaining waiver of consent would allow us to avoid the potential selection/volunteer bias for inclusion of patients particularly interested in screening that can occur when consent is required. Since our main objective is to understand the potential influence varying outreach strategies on subject behavior, we believe that obtaining consent would compromise our primary objective. We have received waiver of consent for similar studies related to cancer screening outreach in the past.

Verbal consent will be obtained from the subsample with whom we plan to conduct post-intervention interviews. A randomly selected sub-sample of ~~200~~ **~~60~~ 134** patients (~~100~~ **~~30~~ approximately 67** from each intervention arm) will be called by the research staff to complete a questionnaire over the phone 6 months after initial outreach was mailed. No more than three phone call attempts will be made to reach the patients. The subjects will be informed about the purpose of the phone call, asked if they would like to participate and if the phone call can be recorded. The phone interview will consist of questions about patient experience, how to improve patient experience, and perception of the impact of HCC surveillance outreach (please see attached phone script). Since these interviews will be conducted over the phone, verbal consent will be obtained and recorded in RedCap. All recordings will be de-identified and stored on the Innovation Center secure drive. Once the de-identified recordings have been transcribed, the audio recording will be deleted. The de-identified transcripts will be stored on the Innovation Center secure drive.

Additionally, a second study will sample a separate sub-set of patients for an interview about their participation in this research study without their consent. This will be submitted under a separate protocol (protocol #TBD).

<u>1.2 Children and Adolescents</u>
None
<u>1.3 Adult Subjects Not Competent to Give Consent</u>
Waiver of consent is being requested.

Waiver of Consent

<u>1.1 Minimal Risk</u>

The study involves no more than minimal risk to subjects and involves no procedures for which written consent is normally required outside of the research context. Surveillance ultrasound are clinically available and utilized tests used to screen for HCC. The research related activity is the randomization of subjects to different outreach strategies and providing them with orders to help complete surveillance imaging.

1.2 Impact on Subject Rights and Welfare

Subjects rights and welfare will not be adversely affected by the waiver of authorization and consent. All subjects will have the opportunity to voluntarily participate in HCC screening. Each arm has the opportunity to engage in HCC screening through routine care as well.

1.3 Waiver Essential to Research

We believe that we would not be able to practically conduct the research without waiver of consent. If we had to obtain either written or verbal consent ahead of time, it would substantially limit our study population and it may alter their participation in the intervention. Thus, we would only learn about the response rate for patients who we were able to speak to for consent. This, would limit the generalizability to practice. Obtaining waiver of consent would allow us to avoid the potential selection/volunteer bias for inclusion of patients particularly interested in screening that can occur when consent is required. Since our main objective is to understand the potential influence varying outreach strategies on subject behavior, we believe that obtaining consent would compromise our primary objective. We have received waiver of consent for similar studies related to cancer screening outreach in the past.

1.4 Additional Information to Subjects

Subjects will be sent a letter explaining that we are offering a special program to patients overdue for ultrasound. Subjects will receive information about the risks and benefits of surveillance ultrasound for HCC screening. Providers will disclose screening results directly to patients if completed.

Potential Study Risks

The risks associated with this study are no more than minimal. Loss of confidentiality is possible, but unlikely. We will minimize this risk by using de-identified information whenever possible and by maintaining all identifiable information on a secure drive and/or in a HIPAA-compliant system (e.g. REDCap). There is also the risk of psychological harm associated with being screened for cancer. This risk will be minimized by the timely communication of screening test results to the subject and the facilitation of follow up diagnostic testing as needed (as is usual practice for screening outreach programs).

Potential Study Benefits

If a participant completes HCC surveillance screening, which is standard of clinical care, the subjects will potentially benefit from participation by increasing the chances of identifying hepatocellular carcinoma at an early stage. Information from this study may benefit society through a better understanding of how to effectively increase overall participation rates in HCC surveillance according to guidelines.

Data and Safety Monitoring

Safety will be monitored on an ongoing basis by the PI and the study team. The PI or designee will review the study charts to evaluate events at each subject interaction to ensure the grade, relationship to the study procedure, expectedness and the course of action for each subject is documented. We will also participate in a Data Safety and Monitoring Board through NIH and the Penn/CHIBE Roybal pilot program.

Risk / Benefit Assessment

The risks associated with this study are no more than minimal. Better knowledge of how to increase mailed screening could potentially address one of the major barriers of accessing care, i.e. having patients come in for clinical office visits. The Principal Investigator believes that the risks of participating in the study are outweighed by the potential benefits of participating in the study.

Summary of Protocol Changes Modifications LOG

Protocol: Increasing surveillance rates for hepatocellular carcinoma among cirrhotic patients
University of Pennsylvania Principal Investigator: Shivan Mehta, MD

Date of Submission	Description of Modification	Rationale for Modification	Approval Date
11/14/19	Initial submission		1/16/20
7/15/20	Clarify procedures<br>Change to study documents<br>Update eligibility criteria<br>Add study personnel	1. Clarify the timing of the data pulls and use of View-only Care Everywhere Encounters<br>2. Update the Mailed Letter Templates<br>3. Add Shivani Kastuar, MD, Internal Medicine PGY2 as Key Study Personnel<br>4. Add other liver carcinomas to exclusion criteria<br>5. Add Mailed Reminder Letter Templates	8/19/20
8/24/20	Update the eligibility criteria	1. Add currently followed at Penn to the inclusion criteria<br>2. Add history of liver transplant and future screening scheduled to exclusion criteria	8/28/20
10/2/20	Update the eligibility criteria<br>Change to study documents	3. Add advanced fibrosis to eligibility criteria<br>4. Expand eligibility criteria to include patients with 1 or more visits with GI in the past 2 years<br>5. Expand exclusion criteria to exclude patients who completed screening in the last 7 months<br>6. Add different screening modality recommended to exclusion criteria<br>7. Update the Mailed Letter Templates	10/8/20
10/21/20	Add and remove study personnel	1. Add Kiernan McNelis, Research Coordinator, as Key Study Personnel<br>2. Remove Jessica Sung from Key Study Personnel	10/29/20

11/20/20	Clarify subject compensation	1. Clarify that Greenphire ClinCard will be used for subject compensation	11/23/20
4/21/21	Clarify outcomes and analysis plan<br>Update study design and procedures<br>Change to study documents	1. Add the evaluation of differences by provider/specialty, incidental findings during imaging, and differences among MyPennMedicine users and non-users to secondary outcomes and analysis plan<br>2. Add third batch to study design<br>3. Update Post-Intervention Interview Script and reduce number of participants included to 60<br>4. Add plan for secondary interview study with a separate sub-set of patients about waiver of informed consent to be submitted under a separate protocol in collaboration with Samantha Stein and Justin Clapp<br>5. Update formatting of Mailed Letter Templates<br>6. Clarify reminder letter procedures	4/22/21
5/6/21	Deviation	The following patients were identified as not meeting study eligibility criteria after outreach was mailed:<br>• 1 patient was found to have completed screening prior to the study window<br>• 2 patients did not have at least one visit with Penn GI in the past 2 years from the date of initial chart review<br>• 1 patient no longer follows with Penn GI, only came to Penn for a second opinion<br>• 1 patient was found to not have definitive evidence of cirrhosis/advanced fibrosis<br><br>These deviations were identified during the 2 month reminder chart review.	6/2/21
6/9/21	Clarify post-intervention randomization plan<br>Update to study documents<br>Add study personnel	1. For the post-intervention phone interviews, a proportion of the 60 subjects will be randomly selected from each batch of randomized participants	6/17/21

		mailed outreach, based on batch size, using STATA.<br>2. Update Post-Intervention Interview Script<br>3. Add Hamzah Shaikh as Key Study Personnel	
7/20/21	Update sample size for post-intervention interviews<br>Update study documents	1. Due to a lower response rate than expected, the sample size for the post-intervention interviews was increased to 134 patients.<br>2. Update Post-Intervention Interview Script	7/23/21
8/11/21	Update study personnel	1. Remove Hamzah Shaikh and Kiernan McNelis as Key Study Personnel<br>2. Add Evelyn Okorie, MS as Key Study Personnel	8/19/21
12/1/21	Deviation	It was discovered that there was a technical error during the Batch 3 ClinCard bulk registration and payment upload that resulted in no funds being processed onto the ClinCards mailed to all 33 of the incentive arm patients in batch 3. The corrective action plan included mailing the affected participants a new ClinCard with an IRB-approved letter apologizing for any confusion.	1/7/22
2/23/22	Deviation	The following patients were identified as not meeting study eligibility criteria after outreach was mailed:<br>• 10 patients were found to have completed screening prior to the study window<br>• 5 patients were found to have severe comorbidities or were on hospice<br><br>These deviations were identified during the final outcomes chart review.	3/25/22

Initial Statistical Analysis Plan

Analysis Plan

1.1 Power and Sample Size

Approximately 860 potentially eligible subjects will be identified via a data abstraction by the Clarity database. Based on preliminary review, we estimate that 30% of patients complete screening outside of Penn or will not meet eligibility criteria per chart review, leaving approximately 600 eligible patients. As such, we anticipate we will have enough patients to enroll at least 600 subjects (and randomize in a 1:2:2 ratio to usual care, opt-out, and incentive arms). We estimate a base return rate for the usual care arm to be 10%. We will consider a meaningful increase in response rate to be 13 percentage points for the opt-out arm as compared to usual care, and 13 percentage points for the incentive arm as compared to the opt-out arm. This will be sufficient sample size using a two-tailed chi-square test of proportions with 80% power and a Type 1 error rate of .025, accounting for two pairwise comparisons with Bonferroni correction (.05/2 = .025).

1.2 Data analysis

We will conduct a chi-squared test of proportions analysis using Stata to compare arm 2 to arm 1 and arm 3 to arm 2 separately using intent-to-treat protocol for the ultrasound completion and any HCC surveillance imaging completion. As exploratory analyses, we will evaluate response by age, gender, race/ethnicity, income at the level of zip code, and etiology of cirrhosis. Analysis will be conducted at least six months after initial outreach.

Qualitative analysis of the post-intervention phone interviews will be conducted using NVivo. This will include thematic analysis of patient experience with the intervention and screening process.

Analysis will be conducted by blinded members of the research team at least 6 months after the mailings have been sent.

Final Statistical Analysis Plan

****New changes from initial protocol notated in bold, parts removed from initial protocol notated in strikethrough**

Analysis Plan

1.1 Power and Sample Size

Approximately 860 potentially eligible subjects will be identified via a data abstraction by the Clarity database. Based on preliminary review, we estimate that 30% of patients complete screening outside of Penn or will not meet eligibility criteria per chart review, leaving approximately 600 eligible patients. As such, we anticipate we will have enough patients to enroll at least 600 subjects (and randomize in a 1:2:2 ratio to usual care, opt-out, and incentive arms). We estimate a base return rate for the usual care arm to be 10%. We will consider a meaningful increase in response rate to be 13 percentage points for the opt-out arm as compared to usual care **(23%)**, and 13 percentage points for the incentive arm as compared to the opt-out arm **(36%)**. This will be sufficient sample size using a two-tailed chi-square test of proportions with 80% power and a Type 1 error rate of .025, accounting for two pairwise comparisons with Bonferroni correction (.05/2 = .025).

1.2 Data analysis

We will conduct a chi-squared test of proportions analysis using Stata to compare arm 2 to arm 1 and arm 3 to arm 2 separately using intent-to-treat protocol for the ultrasound completion and any HCC surveillance imaging completion. As exploratory analyses, we will evaluate response by age, gender, race/ethnicity, income at the level of zip code, ~~and~~ etiology of cirrhosis**, and provider/specialty. We will also evaluate differences in response rate among MyPennMedicine users and non-users and scheduling modality (MyPennMedicine vs. call). Additionally, we will evaluate the percentage of HCC surveillance images that are abnormal, result in follow-up imaging, result in a diagnosis of HCC and follow-up care, and incidental findings during imaging between the study arms.** Analysis will be conducted at least six months after initial outreach.

Qualitative analysis of the post-intervention phone interviews will be conducted using NVivo. This will include thematic analysis of patient experience with the intervention and screening process.

Analysis will be conducted by blinded members of the research team at least 6 months after the mailings have been sent.

Summary of Statistical Analysis Plan Modifications

4/21/21: Before beginning any analysis, we felt it necessary to revisit outcomes and provide a more detailed/thorough analysis plan to ensure both were as complete as possible.

Appendix A

Mailed Letter Templates

<u>Opt-out Framing – Initial Letter</u>

<Patient Name>

<Patient Address>

Dear <Patient Name>,

Patients with cirrhosis or advanced fibrosis are at risk of developing liver cancer (hepatocellular carcinoma [HCC]). HCC is the leading cause of death and need for liver transplant in the United States for patients with cirrhosis. But, HCC is curable if diagnosed early! Patients don't have symptoms before the cancer becomes advanced, so it is important to get regular (every 6 months) screening ultrasounds. You're still at risk, even without symptoms. By getting screened regularly, you will reduce your chance of dying from HCC.

Our records show that you may be overdue for your liver cancer screening.

Penn Medicine is offering a special program to patients overdue for a screening ultrasound. <u>In this envelope, you will find an order for a future screening ultrasound.</u> To schedule your ultrasound right away, either: call the number listed below for the location most convenient to you or go online at MyPennMedicine.org.

**Perelman Center for Advanced Medicine**	XXX-XXX-XXXX
**Penn Presbyterian Medical Center**	XXX-XXX-XXXX
**Pennsylvania Hospital**	XXX-XXX-XXXX
**Penn Radiology Radnor**	XXX-XXX-XXXX

Penn Radiology is committed to the safety of its patients and staff, and has taken extra precautions to create a safe environment and reduce exposure to COVID-19.

If our records are incomplete and you are up to date on liver cancer screening, please let us know by calling XXX-XXX-XXXX.

You and your clinical team will be notified of your screening test results.

Sincerely,

Penn Gastroenterology and Hepatology

<u>Opt-out Framing + Unconditional Incentive – Initial Letter</u>

<Patient Name>

<Patient Address>

Dear <Patient Name>,

Patients with cirrhosis or advanced fibrosis are at risk of developing liver cancer (hepatocellular carcinoma [HCC]). HCC is the leading cause of death and need for liver transplant in the United States for patients with cirrhosis. But, HCC is curable if diagnosed early! Patients don't have symptoms before the cancer becomes advanced, so it is important to get regular (every 6 months) screening ultrasounds. You're still at risk, even without symptoms. By getting screened regularly, you will reduce your chance of dying from HCC.

Our records show that you may be overdue for your liver cancer screening.

Penn Medicine is offering a special program to patients overdue for a screening ultrasound. <u>In this envelope, you will find an order for a future screening ultrasound.</u> To schedule your ultrasound right away, either: call the number listed below for the location most convenient to you or go online atMyPennMedicine.org.

**Perelman Center for Advanced Medicine**	XXX-XXX-XXXX
**Penn Presbyterian Medical Center**	XXX-XXX-XXXX
**Pennsylvania Hospital**	XXX-XXX-XXXX
**Penn Radiology Radnor**	XXX-XXX-XXXX

Penn Radiology is committed to the safety of its patients and staff, and has taken extra precautions to create a safe environment and reduce exposure to COVID-19.

To make things easier, we've also included a $20 gift card. You're welcome to use it however you'd like.

If our records are incomplete and you are up to date on liver cancer screening, please let us know by calling XXX-XXX-XXXX.

You and your clinical team will be notified of your screening test results.

Sincerely,

Penn Gastroenterology and Hepatology

<u>Opt-out Framing – Reminder Letter</u>

<Patient Name>

<Patient Address>

Dear <Patient Name>,

A few weeks ago, we sent you a letter about the importance of completing a screening ultrasound for hepatocellular carcinoma (HCC), a type of liver cancer. Patients with cirrhosis or advanced fibrosis are at risk of developing HCC, so screening is recommended every 6 months.

To date, our records show that you have not been screened.

Penn Medicine is offering a special program to patients overdue for a screening ultrasound. <u>In this envelope, you will find an order for a future screening ultrasound.</u> To schedule your ultrasound right away, either: call the number listed below for the location most convenient to you or go online at MyPennMedicine.org.

**Perelman Center for Advanced Medicine**	XXX-XXX-XXXX
**Penn Presbyterian Medical Center**	XXX-XXX-XXXX
**Pennsylvania Hospital**	XXX-XXX-XXXX
**Penn Radiology Radnor**	XXX-XXX-XXXX

Penn Radiology is committed to the safety of its patients and staff, and has taken extra precautions to create a safe environment and reduce exposure to COVID-19.

If our records are incomplete and you are up to date on liver cancer screening, please let us know by calling XXX-XXX-XXXX.

You and your clinical team will be notified of your screening test results.

Sincerely,

Penn Gastroenterology and Hepatology

<u>Opt-out Framing + Unconditional Incentive – Reminder Letter</u>

<Patient Name>

<Patient Address>

Dear <Patient Name>,

A few weeks ago, we sent you a letter about the importance of completing a screening ultrasound for hepatocellular carcinoma (HCC), a type of liver cancer. Patients with cirrhosis or advanced fibrosis are at risk of developing HCC, so screening is recommended every 6 months.

To date, our records show that you have not been screened.

Penn Medicine is offering a special program to patients overdue for a screening ultrasound. <u>In this envelope, you will find an order for a future screening ultrasound.</u> To schedule your ultrasound right away, either: call the number listed below for the location most convenient to you or go online atMyPennMedicine.org.

**Perelman Center for Advanced Medicine**	XXX-XXX-XXXX
**Penn Presbyterian Medical Center**	XXX-XXX-XXXX
**Pennsylvania Hospital**	XXX-XXX-XXXX
**Penn Radiology Radnor**	XXX-XXX-XXXX

Penn Radiology is committed to the safety of its patients and staff, and has taken extra precautions to create a safe environment and reduce exposure to COVID-19.

If you haven't used it already, remember your $20 gift card can be used anywhere MasterCard is accepted.

If our records are incomplete and you are up to date on liver cancer screening, please let us know by calling XXX-XXX-XXXX.

You and your clinical team will be notified of your screening test results.

Sincerely,

Penn Gastroenterology and Hepatology

Appendix B

Telephone Verbal Consent Script for Post-Intervention Phone Survey

If leaving a message:

Hello, this message is for Mr./Ms. (patient name). This is (research staff name) from the Gastroenterology & Hepatology division at Penn Medicine. I am calling to conduct a brief survey to learn more about patient experience. We will try to reach you again at another time, or call us back at XXX-XXX-XXXX and leave a message with the best time for us to reach you and we'll do our best to call you then. Thank you!

If patient answers:

Hello, my name is (research staff name). I am calling from the Gastroenterology & Hepatology division at Penn Medicine on behalf of Dr. [doctor's name]. Is this (patient name)?

We are conducting a survey to learn more about your most recent experience with care for your liver disease. It will take approximately 10-15 minutes to complete this survey, and your participation is completely voluntary. Are you interested in participating?

If yes, Thank you for agreeing to participate! This call will be recorded to accurately keep track of the feedback you provide, but your responses will be confidential. Loss of confidentiality is possible, but unlikely. We will minimize this risk by using de-identified information and by maintaining all information on the University's secure drive. You may stop the interview at any time without any consequence to you. Do you agree to this phone call being recorded?

If no,

Thank you so much for your time, and have a wonderful rest of your day.

If yes,

The recording will begin now. Thank you so much for agreeing to participate in this survey.

Hepatocellular carcinoma, or HCC for short, is a type of liver cancer, and patients with cirrhosis or advanced fibrosis are at an increased risk of developing this disease. Early detection is very important for HCC because it is curable if diagnosed and treated at an early stage. Because of this, regular HCC screening is recommended for all patients with cirrhosis.

1. Have you completed hepatocellular carcinoma screening (HCC) in the past 6 months?
    a. *If clarification needed*, this is for liver cancer screening and involves an ultrasound, MRI, or CT.
    b. *If no,* can you tell me about why you have not completed screening?
        i. What are the barriers that prevent you from completing screening as recommended?
            1. If COVID is the barrier given, prompt for other reasons they may not complete screening as recommended.
        ii. HCC screening is recommended to be completed every 6 months for all patients with cirrhosis. Do you have a plan to complete this screening in the next few months? Why or Why not?
            1. Please call XXX-XXX-XXXX to schedule your screening.

c. *If yes,* That's great! Congratulations on being up to date on your screening.
    i. Did you complete your screening at Penn Medicine?
        1. Can you tell me about your experience with scheduling?
            a. *Potential probes*: Did you schedule in the office, over the phone, or through MyPennMedicine? If on the phone, did you have to wait on hold? How long was it until the next available appointment? Did you need a referral or pre-authorization?
    ii. Do you typically complete screening every 6 months as recommended?
        1. How are you reminded that you are due for screening?

2. Do you recall receiving a letter in the mail reminding you to complete hepatocellular carcinoma (HCC) screening? (Date letter sent: XXXXX)
    a. What do you remember most about the letter?
    b. Was this reminder helpful for you? How or why not?
    c. What would have made it better or more useful for you?
    d. How would you like to be reminded about these screenings in the future?
        i. Mailed letter? MyPennMedicine? Text? Phone call? Etc.
        ii. Can you tell us why you prefer one over the other?
        iii. When is a good time to get the reminder? How far in advance?
    e. *If patient was in the incentive arm*,
        i. How did you use the gift card we provided for you?
        ii. How did you feel about receiving a gift card from Penn Medicine to help complete screening?
    f. What one or two things would be most helpful in ensuring you complete your screening as recommended, every 6 months?
3. Do you have any other feedback that you would like to give about your experience with HCC screening?

Thank you so much for taking the time to speak with me today. Your feedback is very important to us and will help us improve patient experience in the future. If you have any further questions about this survey, you can reach the study team at XXX-XXX-XXXX. I hope you enjoy the rest of your day. Goodbye.
