# Supplementary material for: A randomized trial of mailed outreach with behavioral economic interventions to improve liver cancer surveillance
Source: Hepatol Commun. 2023 Dec 15;8(1):e0349. doi: 10.1097/HC9.0000000000000349 (PMC10727671; doi:10.1097/HC9.0000000000000349)
Supplement: SUPPLEMENTARY MATERIAL [file hc9-8-e0349-s002.docx]

**Supplement**

*Supplement Table 1. Patients excluded from analysis post-randomization*

| **Study Arm^1^** | **Prior to Outreach,** N (%)^2^ | **Post-Outreach,** N (%)^3^ | **Total Excluded,** N (%)^4^ |
| --- | --- | --- | --- |
| **Usual Care**  (N=123) | 5 (4.0) | 2 (1.6) | 7 (5.7) |
| **Letter+Order**  (N=245) | 11 (4.5) | 10 (4.1) | 21 (8.6) |
| **Letter+Order+Incentive**  (N=247) | 17 (6.9) | 8 (3.2) | 25 (10.1) |
| **Total**  (N=615) | 33 (5.4) | 20 (3.3) | 53 (8.6) |

^1^ N for each arm is total patients randomized.

^2^ P=0.387 using 3x2 chi-square test of proportions.

^3^ P=0.456 using 3x2 chi-square test of proportions.

^4^ P=0.359 using 3x2 chi-square test of proportions.

*Supplement Table 2. Proportion of patients (all randomized) completing abdominal ultrasound in the 6-month period following initial outreach.*

|  | **Completion**  N (%; 95% CI) | **Usual Care vs**  **Letter+Order** | | **Letter+Order vs Letter+Order+Incentive** | |
| --- | --- | --- | --- | --- | --- |
|  |  | **Difference**  % (95% CI) | **p-value^1^** | **Difference**  % (95% CI) | **p-value^1^** |
| **Usual Care**  (N=123) | 34 (27.6; 19.7 to 35.5) |  |  |  |  |
| **Letter+Order**  (N=245) | 131 (53.5; 47.2 to 59.7) | 25.8 (15.7 to 35.9) | <0.001 |  |  |
| **Letter+Order+Incentive**  (N=247) | 130 (52.6; 46.4 to 58.9) |  |  | 0.8 (-8.0 to 9.7) | 0.85 |

^1^ p-value of <.025 was the threshold for statistical significance using the Bonferroni correction for multiple comparisons (.05/2)

*Supplement Figure 1. Subgroup analysis of opt-out vs usual care comparison*

*Supplement Figure 2. Subgroup analysis of incentive vs opt-out comparison*
