# Supplementary figures and images for: A randomized trial of mailed outreach with behavioral economic interventions to improve liver cancer surveillance
Source: Hepatol Commun. 2023 Dec 15;8(1):e0349. doi: 10.1097/HC9.0000000000000349 (PMC10727671; doi:10.1097/HC9.0000000000000349)

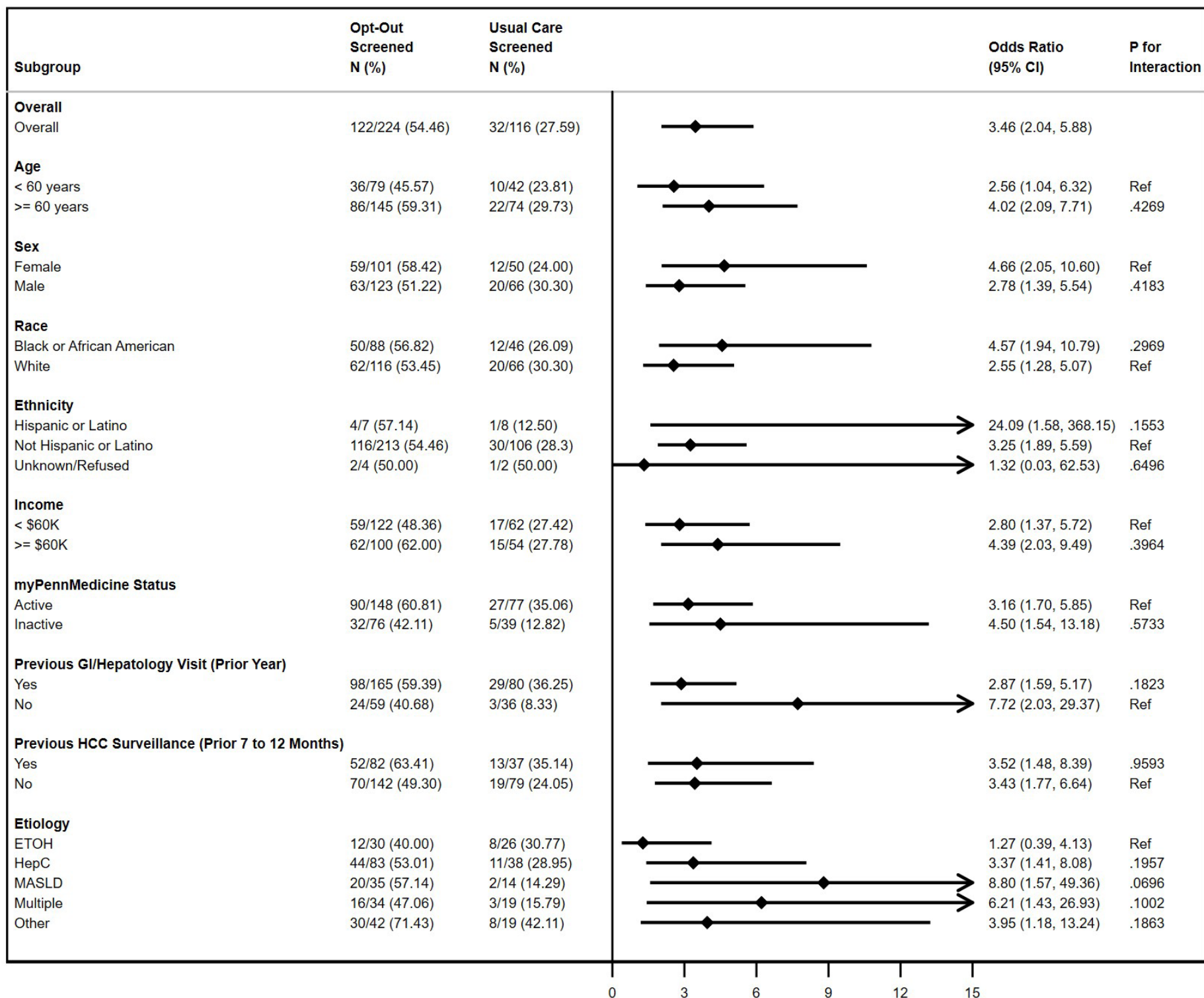

Supplement Figure 1. Subgroup analysis of opt-out vs usual care comparison

Supplement: SUPPLEMENTARY MATERIAL [file hc9-8-e0349-s003.pdf]

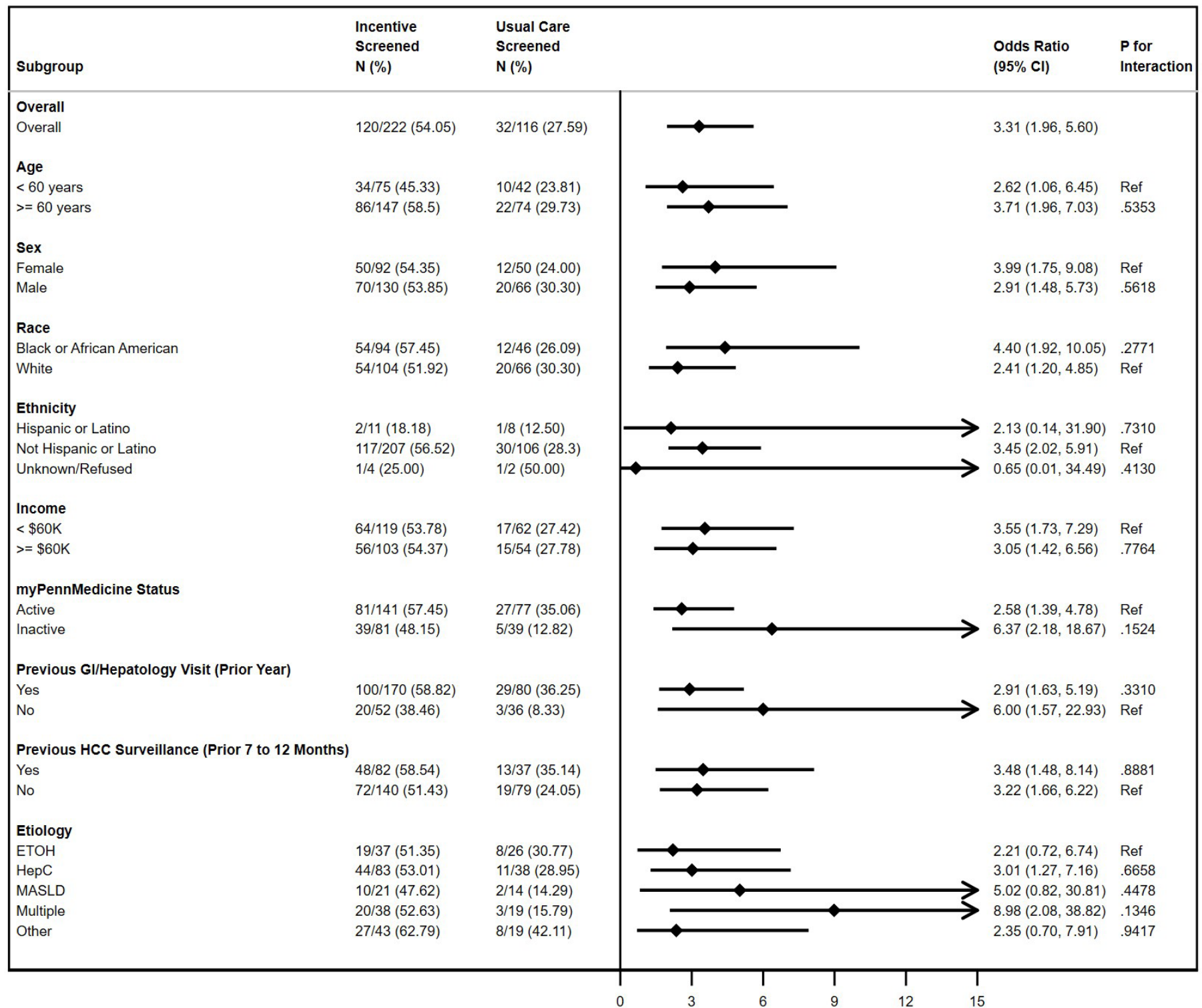

Supplement Figure 2. Subgroup analysis of incentive vs opt-out comparison

Supplement: SUPPLEMENTARY MATERIAL [file hc9-8-e0349-s004.pdf]
